# Multiple Pairwise Analysis of Non-homologous Centromere Coupling Reveals Preferential Chromosome Size-Dependent Interactions and a Role for Bouquet Formation in Establishing the Interaction Pattern

**DOI:** 10.1371/journal.pgen.1006347

**Published:** 2016-10-21

**Authors:** Philippe Lefrançois, Beth Rockmill, Pingxing Xie, G. Shirleen Roeder, Michael Snyder

**Affiliations:** 1 Department of Molecular, Cellular and Developmental Biology Yale University New Haven, United States of America; 2 Faculty of Medicine University of Montreal, Montreal, CANADA; 3 Department of Molecular and Cell Biology University of California Berkeley, Berkeley, United States of America; 4 Faculty of Medicine McGill University Montreal, CANADA; 5 Department of Genetics Stanford University School of Medicine Stanford, United States of America; The University of North Carolina at Chapel Hill, UNITED STATES

## Abstract

During meiosis, chromosomes undergo a homology search in order to locate their homolog to form stable pairs and exchange genetic material. Early in prophase, chromosomes associate in mostly non-homologous pairs, tethered only at their centromeres. This phenomenon, conserved through higher eukaryotes, is termed centromere coupling in budding yeast. Both initiation of recombination and the presence of homologs are dispensable for centromere coupling (occurring in *spo11* mutants and haploids induced to undergo meiosis) but the presence of the synaptonemal complex (SC) protein Zip1 is required. The nature and mechanism of coupling have yet to be elucidated. Here we present the first pairwise analysis of centromere coupling in an effort to uncover underlying rules that may exist within these non-homologous interactions. We designed a novel chromosome conformation capture (3C)-based assay to detect all possible interactions between non-homologous yeast centromeres during early meiosis. Using this variant of 3C-qPCR, we found a size-dependent interaction pattern, in which chromosomes assort preferentially with chromosomes of similar sizes, in haploid and diploid *spo11* cells, but not in a coupling-defective mutant (*spo11 zip1* haploid and diploid yeast). This pattern is also observed in wild-type diploids early in meiosis but disappears as meiosis progresses and homologous chromosomes pair. We found no evidence to support the notion that ancestral centromere homology plays a role in pattern establishment in *S*. *cerevisiae* post-genome duplication. Moreover, we found a role for the meiotic bouquet in establishing the size dependence of centromere coupling, as abolishing bouquet (using the bouquet-defective *spo11 ndj1* mutant) reduces it. Coupling in *spo11 ndj1* rather follows telomere clustering preferences. We propose that a chromosome size preference for centromere coupling helps establish efficient homolog recognition.

## Author Summary

Meiosis enables sexual reproduction in eukaryotes by producing gametes. In the process, it increases genetic diversity through recombination of homologous chromosomes from the parents. Genetic diversity constitutes an evolutionary advantage. Prior to finding their unique pairing partner (homolog), chromosomes associate non-homologously with other chromosomes through their centromeres, a process termed centromere coupling. Little is known about the nature and mechanism of centromere coupling. In this study, we present the first pairwise characterization of this process conserved amongst eukaryotes, using the budding yeast as a model. We quantitatively analyzed the interactions between centromeres for each pair of chromosomes. We observed an interaction pattern based on chromosome size, where centromeres from smaller chromosomes frequently associated with those from other small chromosomes, and a similar association for large chromosomes. This pattern appears ubiquitous, since recombination-defective diploid cells, haploid cells forced to undergo meiosis, and wild-type yeast early in meiosis, until homologous chromosomes become paired, all undergo non-homologous centromere coupling. Centromeres derived from a common ancestor, prior to genome duplication, do not associate more often, excluding ancestral homology as the mechanism. Data from mutants affecting the meiotic bouquet, where all chromosome ends become embedded and clustered in the nuclear envelope prior to coupling, suggest a potential mechanism to generate interactions. Deciphering the mechanisms for proper pairing of homologous chromosomes helps us to understand and prevent chromosomal abnormalities in pregnancy.

## Introduction

Processes in meiosis are geared to recombine homologous chromosomes to both increase genetic diversity, and segregate them efficiently thus producing viable gametes for sexual reproduction. In the absence of recombination (as in a *spo11* diploid cell [1]), chromosomes fail to homologously align, yet the two chromosomal divisions still occur generating highly aneuploid spores. Homologous pairing and recombination between chromosomes favor the formation of stable pairs [2, 3], which are secured by the proteinaceous synaptonemal complex (SC), containing ZMM proteins such as Zip1 [4]. In addition to holding homologs in alignment during meiotic prophase I, the SC is also implicated in crossover formation [5].

Two dynamic homology-independent events precede homolog pairing: the meiotic bouquet and non-homologous centromere coupling. The meiotic bouquet is formed through clustering of telomeres, when they become embedded in a small section of the nuclear envelope [6, 7]. The bouquet persists when meiotic cohesin Rec8 is absent [8]. The bouquet represents a transition from a Rabl configuration, with clustered centromeres close to spindle pole body, to a reverse Rabl configuration during the bouquet stage. The bouquet undergoes rapid telomere-led movements requiring Ndj1 [9, 10], as well as Csm4, Mps3, and actin [11–13]. Bringing telomeres to the nuclear envelope is accomplished mostly by Ndj1 [14], while clustering and rapid movements are more Csm4-dependent [11, 14]. Rapid prophase movements have been shown to destabilize recombination [11] and to contribute to the generation of heterologous and homologous collisions between centromeres for pairing [15]. During the second homology-independent event prior to homolog pairing, “centromere couples” are formed by the transient association of non-homologous chromosomes at their centromeres [16, 17]. Couples are dispersed throughout the nucleus at this stage [16], and an uncoupling mechanism must exist to ensure homolog pairing ensues; a likely candidate for such mechanism is the phosphorylation state of the SC protein Zip1 [18]. The non-homologous centromere associations are proposed to provide a path for a chromosome to find its homolog, as transient non-homologous couples are replaced by stable homologous pairs as pairing, recombination and SC formation progress in a timely fashion [16]. Meiotic non-homologous centromere associations have been described in many model organisms, including yeasts, flies, plants and mammals [19]. In mice, the inability to observe complete coupling suggests that it might be either very short-lived or partial [20, 21].

Studies of centromere coupling in *Saccharomyces cerevisiae* have demonstrated its independence on recombination (as in a *spo11* diploid) and on the presence of homologous chromosomes (as in *spo11* haploids undergoing a forced meiotic induction) [16]. Centromere coupling is dependent on the SC component Zip1 [16, 17] and some requirements regarding the regulation of complete centromere coupling have started to emerge, such as roles for the meiotic cohesin Rec8 [22], for the SC component Zip3 in coupling and tethering [16, 23], and for the phosphorylation of Zip1 by ATM/ATR DSB checkpoint kinases [18]. However, the underlying architecture of centromere coupling remains to be understood. In particular, the presence of an interaction pattern of centromeres, if any, might point towards an intrinsic mechanism for coupling. So far previous studies have relied on low-scale, traditional approaches not amenable to testing this hypothesis on a larger level.

The budding yeast genome, despite its compact size, exhibits a high level of inter-chromosomal contacts and long-range *cis* interactions between distant loci [24]. Chromosome Conformation Capture (3C) enables the detection of DNA regions in close nuclear proximity through formaldehyde crosslinking of such interactions followed by restriction enzyme digestion, dilute ligation to favor intra-molecular products that are crosslinked, and PCR detection [25]. 3C was first developed in budding yeast to study chromosome dynamics during meiosis and higher-order chromatin organization [25], and has since been applied the investigation of diverse biological processes such as silencing [26], organization of the pericentric chromatin [27], and gene looping [28, 29]. 3C has yielded several related techniques that have enabled the characterization of long-range genome associations in mammals [30–34]. One such variant, Taqman-based 3C-qPCR, is well suited for focused studies, with high sensitivity and dynamic range, low background and quantitative detection of interacting fragments [32].

Here we present the first multiple pairwise characterization of centromere coupling. We modified and combined the yeast 3C protocol [35, 36] with Taqman-based real-time detection of 3C ligation products (3C-qPCR) [32] to quantify all possible non-homologous interactions between the 16 centromeres (*CEN*s) of *S*. *cerevisiae* during meiosis. We observed a non-random *CEN* interaction pattern based on similarity of chromosome sizes in strains capable of coupling (*spo11* diploids and haploids), which is absent in coupling-deficient strains (*spo11 zip1* diploids and haploids). Importantly, these size-dependent preferential contacts are present at early time points in normal meiosis (WT diploids), prior to pachytene and full homolog pairing. We also found a role for the meiotic bouquet in pattern establishment, with bouquet absence (*spo11 ndj1*) associated with decreased size dependence. From our results, we propose that centromere coupling, with its preference for chromosomes of similar size, helps chromosomes find their homolog.

## Results/Discussion

### Experimental 3C-qPCR design

**Fig 1:**
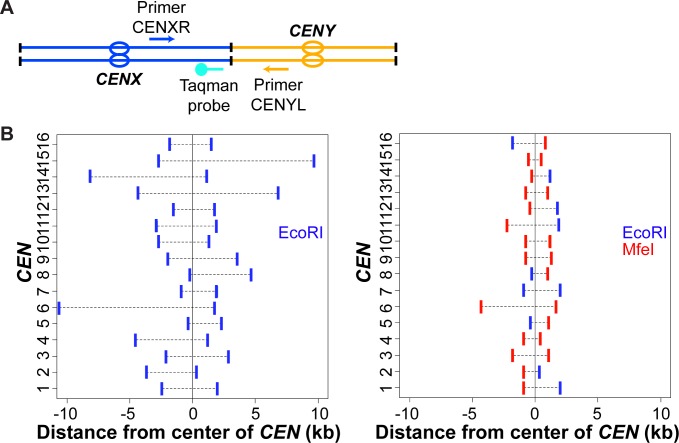
3C2D-qPCR design for characterizing centromere coupling. **(A)** Design of two primers (arrow) and one Taqman probe (ball-and-stick) to quantify the interaction between restriction fragments ligated together, each encompassing a non-homologous centromere (oval). **(B)** Distribution of restriction enzyme sites on fragments encompassing the centromere (*CEN*) on all 16 chromosomes using an EcoRI single digestion (3C) (left) or an EcoRI-MfeI double digestion (3C2D) (right). For each chromosome (on y-axis), the distances of the restriction sites delimitating the *CEN* fragment are given in kilobases (kb), in relation to the center of the *CEN* (x-axis). Blue vertical lines indicate EcoRI sites and red lines indicate MfeI sites.

We used a modified 3C-qPCR assay to specifically look at interactions between non-homologous centromeres. Each of the sixteen similarly-sized centromere regions are defined by restriction enzyme sites. Two primers were designed for each centromere region, one on each side of the restriction fragment oriented towards the enzyme recognition site (Fig 1A). Taqman probes, which allow quantitative detection by real-time qPCR, were synthesized on each side of the *CEN* fragment, closer to the restriction enzyme cutting site than the primer annealing site (Fig 1A). High-Fidelity EcoRI (EcoRI-HF) was used as a suitable restriction enzyme for 3C. However, many EcoRI restriction sites were far from the *CEN* (Fig 1B, left panel; 15/32 sites > 2 kb away from *CEN*), generating fragments which varied in size considerably. Large variations in size might create biases during intra-molecular ligation, favoring the preferential recovery of certain interacting pairs over others [37]. To circumvent this potential issue, we incorporated a double digestion step (3C2D) with the high-fidelity MfeI restriction enzyme (MfeI-HF), generating compatible cohesive ends with EcoRI while recognizing a different consensus site. The 3C2D modification resulted in a more even distribution of restriction site distances from the *CEN*s (Fig 1B, right panel; 2/32 sites > 2 kb away from *CEN*) and centromeric fragments of less variable size (S1 Fig). This experimental design enables the quantification of 480 distinct centromeric interactions, or all 120 possible combinations of non-homologous couples.

To test our 3C2D-qPCR protocol, we isolated genomic DNA from haploid and diploid yeast cells to generate control libraries for 3C, which consist of non-crosslinked, EcoRI-MfeI digested genomic DNA that is randomly ligated [35, 36]. These control samples aim to contain all possible ligation products in near equimolar ratios [25] and serve to test PCR efficiencies of different combinations of primers and Taqman probes [32, 38]. All 480 interactions were compared in haploid and diploid control libraries, looking at the average enrichment (average number of qPCR cycles) across multiple dilutions and replicates for each combination. A majority of combinations have enrichments within 1 qPCR cycle (~2-fold) of the average enrichment across all possible combinations for haploid controls and diploid controls (56% for haploids, 54% for diploids; S2 Fig). For the same combination, there is a high correlation when comparing enrichments between diploid and haploid controls, and 71% lie within 1 qPCR cycle (~2-fold) (Pearson’s r = 0.72, p<10^−15^; S3 Fig). Overall, we find that different combinations of primers and Taqman probes perform similarly.

### Centromere coupling displays chromosome size-dependent interactions

We next analyzed all possible non-homologous *CEN* interactions in a coupling-proficient strain by generating a 3C experimental sample from a *spo11* diploid [16], which consists of EcoRI-MfeI digested, crosslinked chromatin that is ligated in dilute conditions. Non homologous couples in the recombination deficient *spo11* mutants are stable, since homologous pairing does not occur. As a negative control, interactions were also characterized in coupling-deficient *spo11 zip11* diploid [16]. Cells were harvested 14h after meiotic induction, a time point where most *spo11* cells have dispersed the centromere cluster into 16 distinct *CEN* foci (from 32 chromosomes marked by kinetochore component Ctf19) as determined by immunofluorescence microscopy on meiotic chromosome spreads [16, 39]; in *spo11 zip1*, the centromere cluster gives rise to 32 *CEN* foci. In the case of non-homologous centromere coupling, if certain inter-chromosomal centromeric fragments couple more frequently than other combinations, then they would become crosslinked and subsequently ligated at higher frequencies than less-interacting *CEN*s. As a control to ensure that the 3C experimental libraries are enriched for fragments with spatial proximity, we compared amplification of intra-chromosomal proximal fragments (10 kb away) and distal fragments (80 kb away) on the same chromosome (S4A Fig). In the un-crosslinked control libraries, proximal and distal fragments have similar interaction frequencies (randomly-ligated genomic DNA in equimolar proportions) (S4B Fig). For the 3C experimental samples, a higher interaction frequency between proximal fragments than distal fragments is observed (S4B Fig), confirming that we can detect preferential crosslinking and ligation of restriction fragments closer in the nucleus.

**Fig 2:**
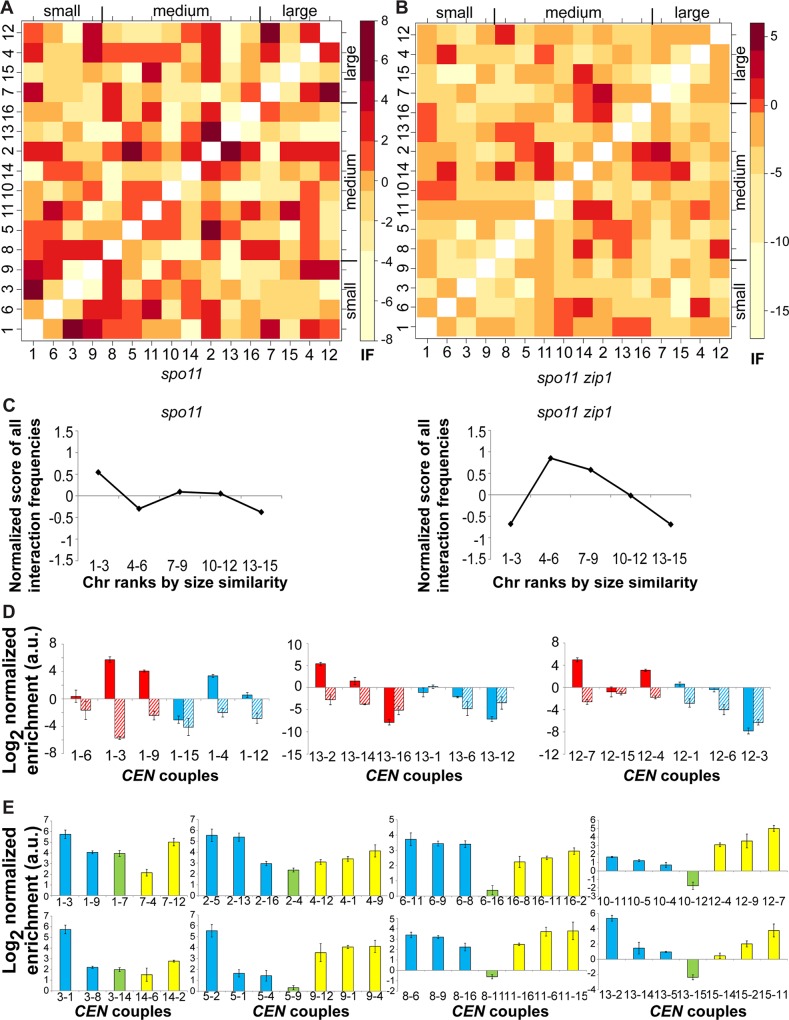
Chromosome size-dependent preferential coupling interactions are present in *spo11* diploids, not in *spo11 zip1* diploids. **(A-B)** Heatmaps of normalized interaction values between non-homologous centromeres in *spo11* **(A)** and *spo11 zip1* **(B)** diploids. Centromeres are arranged from left to right and bottom to top according to their respective chromosome length, from shortest to longest. Darker shades of red indicate a higher level of interaction between non-homologous centromeres. Please note the log2 scale on the color key for interaction frequencies. **(C)** Normalized score of all possible interaction frequencies binned in 5 categories according to chromosome size similarity, in *spo11* and *spo11 zip1* diploids. Using an average level of interaction specific to a particular genotype, a normalized interaction score for the 3 chromosomes most similar in size to one chromosome would be determined. This process would be repeated for all 16 chromosomes, and averaged to a single value representing the bin 1–3. The next 3 closest chromosomes in length would be in bin 4–6, then 7–9, …, up to bin 13–15 for the 3 chromosomes most dissimilar in size. **(D)** Interaction frequencies in *spo11* (plain) or *spo11 zip1* diploids (barred) between the three chromosomes most similar in size (red) or most dissimilar in size (blue) to either a short (chr. 1; left), medium-sized (chr. 13; middle), or long chromosome (chr. 12; right). The log 2 value of the normalized enrichment ratio is plotted on the y-axis (mean in arbitrary units (a.u.) +/- standard deviation). **(E)** Ancestral centromere homology does not explain interaction pattern. For each pair of centromeres sharing ancestral homology (green bar), the log 2 value of the normalized enrichment ratio in *spo11* diploids is plotted on the y-axis (mean in arbitrary units (a.u.) +/- standard deviation). As a comparison, for each centromere sharing homology, centromeres from other chromosomes with the highest interaction frequencies are plotted on the same scale (blue and yellow bars).

We analyzed 480 non-homologous combinations in a *spo11* diploid and in a *spo11 zip1* diploid using 3C2D-qPCR. Interaction frequencies between non-homologous centromeres were plotted on a heatmap after normalization (Fig 2A for *spo11* diploid and Fig 2B for *spo11 zip1* diploid). For each chromosome, the 15 non-homologous chromosomes were ranked according to the strength of their *CEN* interaction (S5 Fig for *spo11* diploid and S6 Fig for *spo11 zip1* diploid). In the case of the *spo11* diploid library, we observed a non-random interaction pattern during centromere coupling, with centromeres of smaller chromosomes interacting preferentially with those from small chromosomes (Fig 2A and S5 Fig). In brief, centromeres interact with centromeres from liked-size chromosomes more frequently. To test the significance of this relationship, we asked the following: do the top three *CEN*s with the highest interacting frequencies happen to be the three closest chromosomes in length more often than random? By performing a non-parametric permutation test to generate a randomized matrix, representing what level of association is expected by chance alone, we found that this chromosome size interaction pattern was present in coupling-proficient *spo11* diploids (p < 0.01), but not in coupling-defective *spo11 zip1* diploids (p > 0.10). We plotted a normalized interaction score of all possible interaction frequencies, binned in 5 categories according to chromosome size similarity (bin 1–3 for the 3 chromosomes most similar in size, …, bin 13–15 for the 3 chromosomes most dissimilar in size). A positive value indicates an increased frequency of interactions compared to the average level of interaction for that particular genotype, and a negative value indicates fewer interactions than average. Couples between one chromosome and its three chromosomes most similar in size (bin 1–3) are overrepresented in *spo11* diploids, but not in *spo11 zip1* diploids (Fig 2C). Examples for coupling partners most similar or dissimilar in size to a short, medium and large chromosome are presented in Fig 2D. In *spo11* diploids, the interaction pattern was identical for the four shortest chromosomes and the four largest chromosomes. We performed a sensitivity analysis for our model, considering a) the three most interacting *CEN*s and the five closest chromosomes in length, b) the five most interacting *CEN*s and the three closest chromosomes in length, and c) the five most interacting *CEN*s and the five closest chromosomes in length. In all cases, the pattern was statistically significant in *spo11* diploids (p < 0.05), but not in *spo11 zip1* diploids (p > 0.10).

We compared the mean raw cycle numbers (+/- standard error of the mean (SEM)) between *spo11* and *spo11 zip1* diploids as an estimate of the total number of interactions, with a smaller cycle value representing a quicker qPCR amplification to a detectable level above background, which is directly related to the abundance of a particular couple among all 3C DNA ligation products. We observed a ~ 24-fold difference between *spo11* diploids (32.64 +/- 0.30) and *spo11 zip1* diploids (37.21 +/- 0.34) (enrichment = difference of 4.57 on a log2 scale). Differences in raw interaction frequencies between *spo11* and *spo11 zip1* diploids are plotted as a heatmap in S7 Fig. These results suggest that coupling is absent in *spo11 zip1*, consistent with previous imaging studies [16, 17]. Although a highly dynamic process, where couples form but are not maintained, cannot be ruled out from these genomic data, it is unlikely.

### Ancestral homology does not contribute to preferential centromere association

Since DNA sequence homology on chromosome arms has a role in homolog pairing [3], preferential interactions between non-homologous couples might derive from large blocks of homologous regions on arms. For all 16 chromosomes of *Saccharomyces cerevisiae*, we considered the extent of homologous regions between pairs *of S*. *cerevisiae* chromosomes that had apparently derived from a single chromosome of the reconstructed yeast ancestor prior to whole genome duplication [40]. Following a similar non-parametric testing procedure to generate a randomized matrix of interaction frequencies, we found that sequence homology on chromosome arms cannot explain the pairwise coupling pattern observed in *spo11* diploids (p > 0.05). It is also possible to align pairs of centromeres with optimal homology based on ancestry by identifying the two *Saccharomyces cerevisiae* centromere regions related to a single centromere region from *Kluyveromyces waltii*, a budding yeast that diverged from the *Saccharomyces* lineage prior to whole-genome duplication [41, 42]. We asked whether ancestral centromere homology was the mechanism for centromere coupling, with the strongest interactions between the two ancestral centromeres (*CEN1*-*CEN7*, *CEN2-CEN4*, *CEN3-CEN14*, *CEN5-CEN9*, *CEN6-CEN16*, *CEN8-CEN11*, *CEN10-CEN12*, *CEN13-CEN15*) [42]. We found that, for each centromere, the strongest interacting partner was never its ancestral sister (Fig 2E).

### Obligated non-homologous couples in haploid yeasts also interact via a chromosome size-dependent pattern

**Fig 3:**
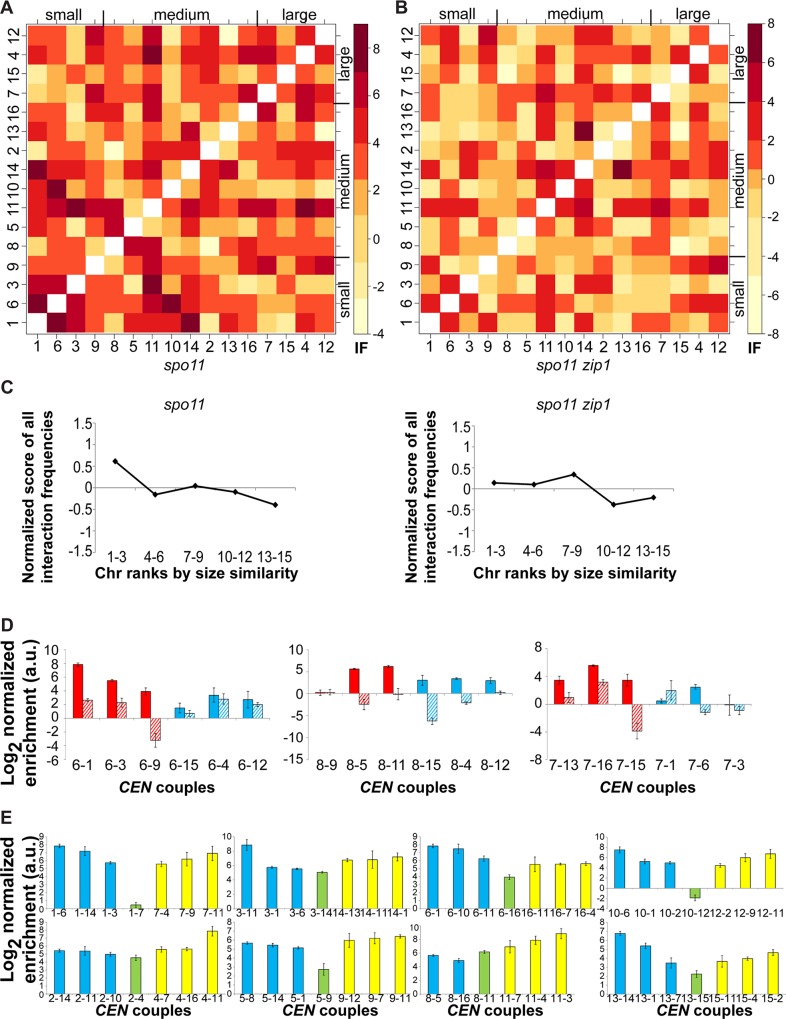
Chromosome size-dependent preferential coupling interactions are present in *spo11* haploids, not in *spo11 zip1* haploids. **(A-B)** Heatmaps of normalized interaction values between non-homologous centromeres in *spo11* **(A)** and *spo11 zip1* **(B)** haploids. Centromeres are arranged from left to right and bottom to top according to their respective chromosome length, from shortest to longest. Darker shades of red indicate a higher level of interaction between non-homologous centromeres. Please note the log2 scale on the color key for interaction frequencies. **(C)** Normalized score of all possible interaction frequencies binned in 5 categories according to chromosome size similarity, in *spo11* and *spo11 zip1* haploids. **(D)** Interaction frequencies in *spo11* (plain) or *spo11 zip1* haploids (barred) between the three chromosomes most similar in size (red) or most dissimilar in size (blue) to either a short (chr. 6; left), medium-sized (chr. 8; middle), or long chromosome (chr. 7; right). The log 2 value of the normalized enrichment ratio is plotted on the y-axis (mean in arbitrary units (a.u.) +/- standard deviation). **(E)** Ancestral centromere homology does not explain interaction pattern. For each pair of centromeres sharing ancestral homology (green bar), the log 2 value of the normalized enrichment ratio in *spo11* haploids is plotted on the y-axis (mean in arbitrary units (a.u.) +/- standard deviation). As a comparison, for each centromere sharing homology, centromeres from other chromosomes with the highest interaction frequencies are plotted on the same scale (blue and yellow bars).

In budding yeast, haploid cells, lacking any homologues, can be forced to undergo a meiotic induction by expressing the opposite mating type cassette from an ectopic locus [6]. Haploids exhibit centromere coupling, forming 8 *CEN* couples that are *de facto* non-homologous, from the 16 chromosomes [16]. As in diploids, coupling is abolished in the absence of Zip1 [16]. We wondered whether preferential interactions during centromere coupling also follow a chromosome size-dependent pattern in the absence of potential interactions with homologous chromosomes. We repeated the multiple pairwise 3C2D-qPCR analysis to detect all possible centromeric interactions in coupling-proficient *spo11* haploids and in coupling-deficient *spo11 zip1* haploids. Cells were harvested 20h after meiotic induction, a time point where most cells contain 8 *CEN* foci (from 16 chromosomes marked by kinetochore component Ctf19) as determined by immunofluorescence microscopy on meiotic chromosome spreads [16, 39]. Interaction frequencies between non-homologous centromeres were plotted on a heatmap after normalization (Fig 3A for *spo11* haploid and Fig 3B for *spo11 zip1* haploid). Again all 15 chromosomes were ranked by the strength of their *CEN* interaction for any given chromosome (S8 Fig for *spo11* haploid and S9 Fig for *spo11 zip1* haploid). Similarly to *spo11* diploids, *spo11* haploids have preferential interactions based on comparable chromosome sizes (Fig 3A and S9 Fig). For each chromosome, there is a strong, non-random, bias towards preferential interactions with chromosomes of similar sizes in *spo11* haploids (top three *CEN*s closest in length) (p < 0.01). Again this pattern was absent in coupling-deficient *spo11 zip1* haploids (p > 0.10) and ancestral centromere homology does not play a role in haploids to establish the interaction pattern (Fig 3E), with only *CEN8* having its strongest interacting partner as its ancestral sister *CEN11*. Note that Chromosome 11 is one of the chromosomes most similar in size to chromosome 8 (667 kb vs. 563 kb, respectively). Using a normalized interaction score of all possible interaction frequencies binned in 5 categories according to chromosome size similarity, couples between one chromosome and its three chromosomes most similar in size (bin 1–3) are overrepresented in *spo11* haploids, but not in *spo11 zip1* haploids (Fig 3C). Examples for coupling partners most similar or dissimilar in size to a short, medium and large chromosome are presented in Fig 3D. Of note, we observed a strong level of interaction with chromosome 11 for both *spo11* and *spo11 zip1* haploids (horizontal line). We do not have a biological explanation for such a pattern. However, when looking at the raw interaction frequency differences between *spo11* and *spo11 zip1* haploids (S10 Fig), this horizontal line became attenuated. Moreover, using a randomization approach, we determined that, in *spo11* haploids, only the four shortest chromosomes had preferential interactions based on size similarity (p < 0.05), but not the four largest chromosomes (p > 0.10).

Similarly to diploids, using the mean raw cycle numbers (+/- SEM) as an estimate of the total amount of interactions, *spo11* haploids had on average ~17-fold more interactions than *spo11 zip1* haploids (30.25 +/- 0.22 for *spo11* haploids vs. 34.30 +/- 0.22 for *spo11 zip1* haploids) (enrichment = difference of 4.05 on a log2 scale). Moreover, haploids displayed more coupling interactions than diploids (~ 5–8 fold difference in cycle numbers), consistent with obligate non-homologous interactions in haploids.

Both *spo11* haploids and diploids have preferential coupling interactions based on similarity in chromosome size. Overall 3C2D-qPCR profiles show a statistically significant agreement, whether using raw interaction frequencies (R^2^ = 0.22; p < 0.001) or ranked data (ρ = 0.27; p < 10^−4^). This agreement between both datasets tends to be greater in the upper third of the lists, for the top 5 interacting pairs (44% overlap, p = 0.01, non-parametric resampling test).

### Chromosome size-dependent interactions is confirmed by immunofluorescence microscopy on meiotic spreads

We then asked whether a strong coupling interaction determined by our genomic approach could be confirmed using immunofluorescence microscopy. According to our 3C2D-qPCR data, two centromeres from small chromosomes, *CEN1* and *CEN3*, would form non-homologous couples preferentially. We tested this possibility by constructing a haploid *spo11* strain with a lacO array at *CEN3*, a TetO array at *CEN1*, LacI-GFP and TetR-mCherry. Meiotic spreads from this strain were used to assess *CEN1/CEN3* colocalization by immunofluorescence. A control chromosome pair was chosen from our genomic data. *CEN3*, from a short chromosome, and *CEN5*, from a medium-sized chromosome, do not appear to interact preferentially above a low-medium background level. We constructed an isogenic strain but with the TetO array at *CEN5* and quantified *CEN3/CEN5* couples by immunofluorescence. Chromosome spreads were first screened with DAPI to ensure proper spreading [39]. Among those spreads, the kinetochore arrangement was assessed with Ctf19 immunostaining for ~8 *Ctf19* foci (centromeres) (Material and Methods) [16].

**Fig 4:**
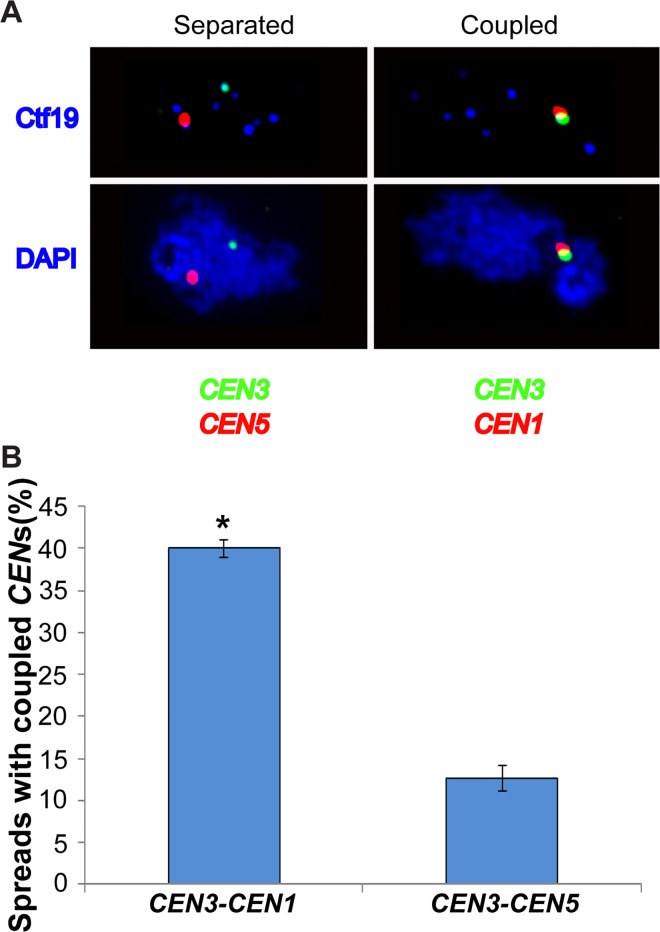
Cytological demonstration of differential coupling from 3C2D-qPCR data. **(A)** Representations of two spread nuclei (from *spo11* haploid strains) in the coupling phase of meiosis. Left: centromeres 3 and 5 are separated (not coupled). Right: centromeres 1 and 3 are coupled. Upper: Red focus is centromere 5 or 1, green focus is centromere 3, and blue foci indicate kinetochores (Ctf19). Lower panels, same as above, but blue marks DNA (DAPI). **(B)** *spo11* haploids containing two centromeres marked with GFP-*CEN3* and either mCherry-*CEN1* (BR5890) or mCherry-*CEN5* (BR5891) were scored for centromere coupling (overlap of red and green signals) in three independent experiments (see Methods for details). The percentage of spreads with coupled *CEN*s is given on the y-axis (mean +/- standard deviation). The asterisk indicates statistical significance (P < 0.01). P-value was calculated using Fisher’s Exact Test (in this case, P < 10^−7^).

As predicted from our 3C2D-qPCR genomic analyses, *CEN3* associates more often with *CEN1* than with *CEN5* on individual meiotic spreads (Fig 4B; p < 10^−7^, Fisher’s Exact Test). Indeed, *CEN1* and *CEN3* were coupled on average in 40% of meiotic spreads (range = 38–42%), while *CEN3* and *CEN5* were coupled on average in 12.7% of spreads (range = 10–16%), a more than 3-fold increase for the predicted interaction between *CEN1* and *CEN3* based on the similarity of their chromosome lengths.

### Size-dependent interactions occur early in meiosis and vanish as meiosis progresses in wild-type diploid yeast

In a wild-type (WT) diploid yeast, non-homologous centromere couples are transient, present early in meiosis and gradually replaced by stable homologous pairs by pachytene [16]. Using our genomic assay, we next asked whether coupling interactions occurring in a wild-type yeast show the same size preference for partners. WT cells were harvested at multiple time points after meiotic induction (8h, 9h, 10h, 11h, and 14h (pachytene)). Immunofluorescence was performed to monitor meiotic progression from centromere organization (Ctf19) and the appearance of SC components (Zip1 and Red1) (S11 Fig). Interaction frequencies between centromeres were plotted on a heatmap after normalization (S12A Fig). For each chromosome, the 15 non-homologous chromosomes were ranked according to the strength of their *CEN* interaction (S12B Fig). Early in meiosis (8h, 9h time points), centromeres interact preferentially based on similarity of chromosome sizes, as found in *spo11* diploids and haploids (S12 Fig; top three chromosomes closest in length: p < 0.01). Later in meiosis this pattern becomes less significant at 10h and 11h (S12 Fig; p > 0.10 for 10h and p = 0.064 for 11h) and achieves its lowest value at 14h/pachytene (S12 Fig; p > 0.10).

**Fig 5:**
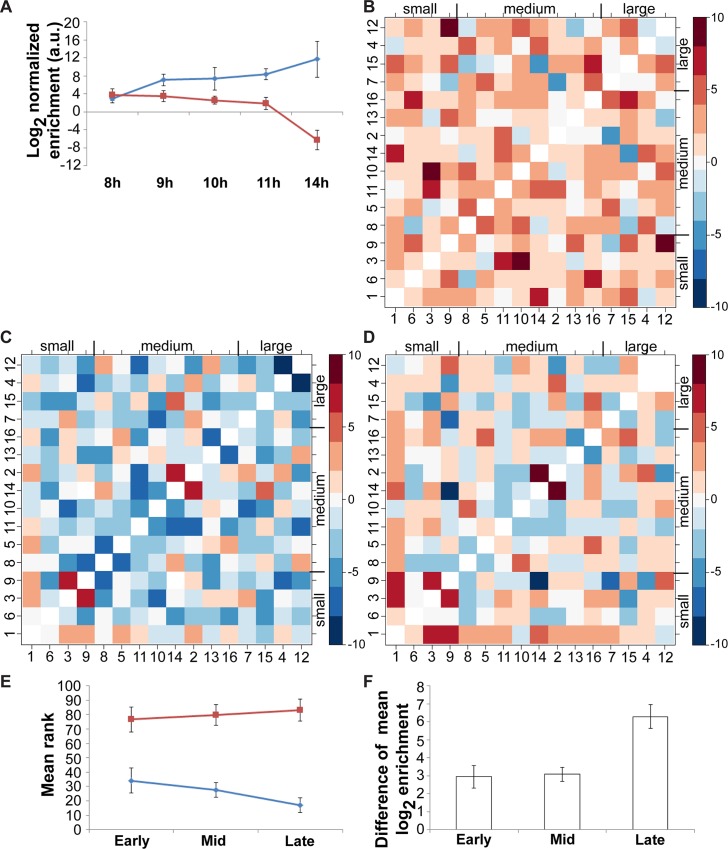
Progression of centromere coupling in wild-type diploid yeast cells. **(A)** Coupling interactions between *CEN1* and *CEN3* (from short chromosomes; blue) are increased while those between *CEN4* and *CEN12* (from long chromosomes; red) are decreased as meiosis progresses (time in hours after meiotic induction on x-axis). Log 2 values of the normalized enrichment ratios are plotted on the y-axis (means in arbitrary units (a.u.) +/- standard deviation). **(B-C-D)** Relative changes in interaction frequencies during meiosis. We grouped 8h and 9h as early, 10h and 11h as mid; 14h was considered late. Differences of normalized interaction frequencies were plotted on a heatmap to compare their relative progression (early→mid **(B)**, mid→late **(C)**, and early→late **(D)**). Heatmaps were unscaled, with white meaning no changes, red for increases, and blue for decreases. Please note the log2 scale on the color key for interaction frequencies. **(E)** Average rank of all interactions involving *CEN1* (from shortest chromosome; blue) and *CEN12* (from longest chromosome; red). The mean rank (out of 120) for the 15 interactions involving each centromere is plotted with its standard deviation, for early, mid and late time points. **(F)** Differences of mean normalized enrichment ratios for all 15 interactions involving *CEN1* and those involving *CEN12* (*CEN1* value minus *CEN12* value). Log 2 values of these differences are plotted on the y-axis (in arbitrary units (a.u.) +/- standard deviation), for early, mid and late time points.

In budding yeast, the shortest chromosomes are the last ones to pair with their homolog and synapse [15]. Extrapolating this result to non-homologous coupling, one would then expect that interactions involving larger chromosomes would decrease and that the proportion of interactions between small chromosomes would increase. As meiosis progresses (from 8h to 14h), our genomic data suggest that this is the case. Indeed, using a randomization procedure, we observed that, for the final time point in our WT yeast (late; 14h), the interaction pattern based on chromosome size is very strong for the four shortest chromosomes (p < 0.01), and completely absent for the four largest chromosomes. For example, interaction frequencies between chromosomes 1 and 3 (short chromosomes) are increased while those between chromosomes 4 and 12 (largest chromosomes) decrease (Fig 5A). For simplifying a comprehensive analysis, we combined meiotic time points 8h and 9h as “early”, and 10h and 11h as “mid”. Time point 14h is considered “late”. Differences of normalized interaction frequencies between non-homologous centromeres were plotted on a heatmap to compare their relative progression (Fig 5B, 5C and 5D for early→mid, mid→late, and early→late; red = relative increase, blue = relative decrease). When considering heatmaps involving the late time point, there is a relative increase in the interactions involving smaller chromosomes, most obvious with chromosome 1, and a relative decrease when longer chromosomes are involved, especially for chromosome 12, albeit less striking in general. Additionally, by ranking interactions frequencies for all 120 unique combinations of couples, i.e. chr1-2…chr1-16, chr2-3 …chr2-16, …, chr15-16 (couple with most interactions = 1, couple with least interactions = 120), we observed that the average rank of all 15 interactions involving chromosome 1 (smallest chromosome) became closer to the first ranks, from 34 (early) to 28 (mid) to 16 (late) (out of 120), while that of all interactions involving chromosome 12 (largest chromosome) became closer to the last ranks, from 77 to 80 to 83 (out of 120) (Fig 5E). This trend towards the top ranked couples for non-homologous interactions with *CEN1* signifies that *CEN1* continues to undergo active coupling. Moreover, we typically observe more coupling interactions for *CEN1* than *CEN12*, with a difference in their mean enrichment ratios approximately 8-fold for early and mid time points (Fig 5F). This difference in mean enrichment ratios jumps from 8-fold to 78-fold at the late time point (Fig 5F), revealing that coupling interactions with smaller chromosomes become more prevalent as meiosis proceeds while non-homologous contacts between large chromosomes decrease. This supports the observation that longer homologous chromosomes pair first [15], presumably because of larger blocks of homology on chromosome arms, or more numerous potential pairing blocks. As such, long chromosomes would be taken out of the coupling pool earlier, leaving mostly short chromosomes engaged in non-homologous interactions, in search for their homolog.

A unique limitation of this experiment, during which coupling is analyzed in wild-type yeast cells, is their asynchronous entry into meiosis. Wild-type yeasts from the BR1919-8B background enter meiosis fairly asynchronously [43]. Our cytological analysis in wild-type cells (S11 Fig) revealed that no more than ~ 75% of the cells at each time point, but more than ~ 50%, are in the same stage. This heterogeneity of cells in different stages likely contributes to noise present in the interaction frequency heat maps, and demand caution in interpreting the results. However, despite the noise resulting from the isolation of cells at different steps in meiosis, we were able to identify an interaction pattern based on size and confirm previous findings about earlier pairing of larger chromosomes [15].

In contrast, asynchronous entry into meiosis is not an issue for the remainder of the experiments performed in a BR1919-8B *spo11* background. In diploid and haploid strains lacking Spo11, centromere coupling persists through prophase for several hours [16, 17]. Studies performed in the same BR1919-8B *spo11* background, at similar time points for cell collection than this study, found that centromeres formed distinct foci in ~ 95% of diploid *spo11* cells and haploid *spo11* cells (~5% of cells with clustered centromeres) [22, 44]. Similarly, aliquots taken as cells were harvested from our large cultures of various *spo11* strains showed that centromeres formed multiple distinct foci (separated/coupled) in > 80% of cells (median 91.4%) (S13 Fig). Thus, in contrast to wild-type BR1919-8B cells, *spo11* BR1919-8B are minimally influenced by asynchronous entry into meiosis, as they remain in a state with centromeres forming distinct foci for an extended period of time.

### Abolition of the meiotic bouquet affects chromosome size-dependent coupling interactions

**Fig 6:**
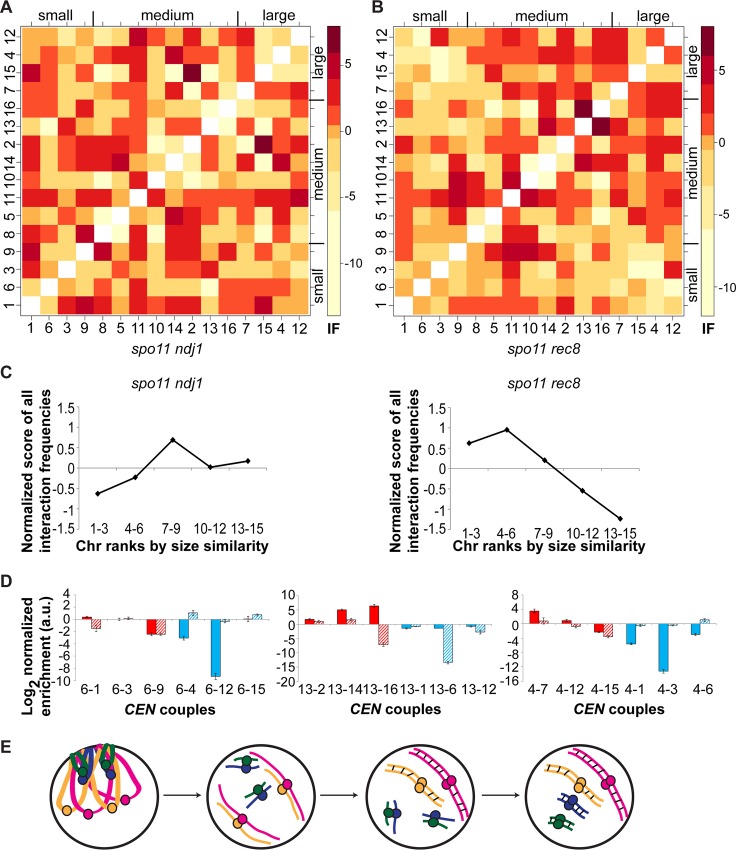
Chromosome size-dependent interaction pattern in meiotic bouquet mutants. **(A-B)** Heatmaps of normalized interaction values between non-homologous centromeres in *spo11 ndj1* **(A)** and *spo11 rec8* **(B)** diploids. Centromeres are arranged from left to right and bottom to top according to their respective chromosome length, from shortest to longest. Darker shades of red indicate a higher level of interaction between non-homologous centromeres. Please note the log2 scale on the color key for interaction frequencies. **(C)** Normalized score of all possible interaction frequencies binned in 5 categories according to chromosome size similarity, in *spo11 ndj1* and *spo11 rec8* diploids. **(D)** Interaction frequencies in *spo11 rec8* (plain) or *spo11 ndj1* diploids (barred) between the three chromosomes most similar in size (red) or most dissimilar in size (blue) to either a short (chr. 6; left), medium-sized (chr. 13; middle), or long chromosome (chr. 4; right). The log 2 value of the normalized enrichment ratio is plotted on the y-axis (mean in arbitrary units (a.u.) +/- standard deviation). **(E)** Model of centromeric interactions during coupling (see Results and Discussion section). Circles depict centromeres and small black lines indicate formation of SC.

Given the chromosome size-dependent preferential interactions we observed, a possible mechanism to help in establishing this interaction pattern could be bouquet formation. Early in zygotene, chromosomes associate non-homologously at their telomeres in a small region of the nuclear envelope, forming the meiotic bouquet [6, 7]. Bouquet formation is disrupted in *ndj1* mutants [7, 9, 10] and persists in *rec8* mutants [8]. Centromere coupling has been previously assessed by microscopy approaches in strains with altered bouquet formation. Bouquet formation was found to be dispensable for centromere coupling, given that *spo11 ndj1* diploids form no bouquet but still had ~16 *CEN* foci, as did coupling-proficient *spo11* diploids [16]. On the other hand, immunofluorescence data suggest that only 23% of *spo11 rec8* diploid cells undergo non-homologous coupling (16–20 *CEN* foci) [22], arguing that *spo11 rec8* diploids display at most partial coupling. The coupling defect observed in *spo11 rec8* diploids is likely due to a reduction in Zip1 loading around centromeres, in particular on cohesin-rich pericentromeric regions [22]. Using the high sensitivity of our 3C2D-qPCR method for assessing specifically non-homologous centromeric interactions, we first tested the hypothesis that the size-dependent pairwise pattern would be absent (or decreased) in bouquet-deficient *spo11 ndj1* diploids. Interaction frequencies between non-homologous centromeres were plotted on a heatmap after normalization (Fig 6A for *spo11 ndj1* diploids). For each chromosome, the 15 non-homologous chromosomes were ranked according to the strength of their *CEN* interaction (S14 Fig for *spo11 ndj1* diploids). Consistent with a role for bouquets in size establishment, the chromosome size-dependent pattern was absent when the bouquet was abolished in *spo11 ndj1* diploids (Fig 6A and S14 Fig; top three chromosomes closest in length: p > 0.10). In normalized interaction score plots, *spo11 ndj1* diploids do not show preference for liked-size chromosomes (Fig 6C). In *spo11 ndj1* diploids, there is a ~38-fold increase in the raw interaction levels estimated by raw cycle numbers compared to *spo11 zip1* diploids (31.95 +/- 0.35 for *spo11 ndj1* vs. 37.21 +/- 0.34 for *spo11 zip1*) (enrichment = difference of 5.26 on a log2 scale), a similar increase as observed in *spo11* diploids, which is consistent with robust coupling [16]. Despite the fact that *spo11 rec8* diploids undergo at most partial coupling, i.e. coupling in a minority of cells [22], we asked whether we could detect non-homologous coupling interactions in those cells, taking advantage of the sensitivity and specificity of our 3C2D-qPCR assay. In *spo11 rec8* diploids, interactions are reduced by ~6–9 fold compared to coupling-proficient strains (35.13 +/- 0.31 for *spo11 rec8* vs. 31.95 +/- 0.35 for *spo11 ndj1* or 32.64 +/- 0.30 for *spo11*) (enrichments = differences of 3.18 and 2.49 on a log2 scale), but are increased ~4-fold compared to *spo11 zip1* (37.21 +/- 0.34) (enrichment = difference of 2.08 on a log2 scale). This is in accordance with previous data showing a defect in coupling in *spo11 rec8* diploids [22]. Similar to *spo11* diploids, *spo11 rec8* diploids show a significant bias towards interactions between chromosomes of similar length (Fig 6B and S15 Fig; top three chromosomes closest in length: p < 0.01). In normalized interaction score plots, looking at bins 1…3 and 4…6, *spo11 rec8* diploids display a robust chromosome size-dependent pattern (Fig 6C). This suggests that the size-dependent pairwise pattern is not disrupted in bouquet-persisting *spo11 rec8* diploids. Uniquely, for *spo11 rec8* diploids, a significant decrease in *CEN* interactions between chromosomes of most dissimilar length (e.g. small vs. large) is seen. To test the significance of this relationship based on dissimilarity of chromosome lengths, we performed a non-parametric permutation test similar to the one previously used for similarity of sizes: do the last three *CEN*s with the lowest interaction frequencies happen to be the three chromosomes most dissimilar in chromosome lengths more often than expected by chance? This avoidance of coupling interactions between chromosomes of most dissimilar lengths was found in *spo11 rec8* diploids (p < 0.01), but not in *spo11*, *spo11 ndj1* or *spo11 zip1* diploids (p > 0.10). Accordingly, normalized interaction score plots depict a strong underrepresentation of interactions between chromosomes of most dissimilar length in *spo11 rec8* (Fig 6C). This trend held true for small, medium-sized and large chromosomes (Fig 6D). Even compared to *spo11* diploids and haploids, *spo11 rec8* diploids show a greater decrease in normalized interaction score across all 16 chromosomes between the three partners most similar in size to a particular chromosome and the three most dissimilar in size (Figs 2C, 3C and 6C; bin 1–3 vs. bin 13–15). However, caution should be exercised in interpreting these results, in light of reduced levels of coupling in *spo11 rec8* diploids ([22], and confirmation by the lower raw interaction frequencies, in this study).

Overall, these results suggest that the meiotic bouquet might create a favorable architecture for assorting chromosomes by length, thus helping to establish non-homologous coupling contacts based on chromosome size. Recent *in silico* simulations have demonstrated that the bouquet sorts chromosomes based on their size [45]. The tightness of the bouquet (i.e. clustering opposite telomeres on a narrower section of the nuclear envelope) plays a greater role for associations between shorter chromosomes, with these chromosomes arranged in a shorter U-shaped structure [45]. In contrast, the levels of chromosomal rigidity/flexibility and of periodic juxtaposition have a greater influence on interactions between longer chromosomes. Absence of the bouquet, as in a *spo11 ndj1* diploid, disrupts the interaction pattern. On the other hand, persistence of the bouquet, as in a *spo11 rec8* strain, does not disrupt the interaction pattern in the minority of cells that undergo coupling in this genotype, and, additionally, we observed avoidance of interactions between *CEN*s from chromosomes of most dissimilar sizes.

In the meiotic bouquet, with telomeres confined to a section of the nuclear envelope, the centromeres of chromosomes likely project towards the center of the nucleus, into a reverse Rabl-like configuration. Since most of the centromeres do not sit precisely at the midpoint of the 16 yeast chromosomes, the length of the shorter arm of the chromosome (centromere to telomere) would limit the distance from the base of the bouquet. As such, centromeres from chromosomes with similarly-sized short arms might be closer than even more similarly-sized chromosomes, hence engaging in coupling interactions more often. For example, in some long chromosomes (12 and 2) the centromeres are subtelocentric and thus might associate more often with very small chromosomes (such as 3, 5 and 6). We repeated our analysis for *spo11* and *spo11 zip1* diploids and haploids, but did not observe any association between the level of interaction frequencies and the similarity of short arm sizes (p > 0.05). Thus physical constraints based on chromosome size, such as 3D conformation, chromosomal condensation and bending rigidity in the arms, probably play a greater role in the establishment of couples than the maximum linear distance from the centromere to its closest telomere. Another contribution to the size-dependent pattern might come from the interplay between centromeres, telomeres and the spindle pole body. In fission yeast, the telomere bouquet is necessary for accurate chromosome segregation through interactions with the spindle pole body and spindle assembly, independent of recombination [46]. Centromeres need to interact with the telomere-spindle pole body microenvironment for full assembly during meiosis [47]. Of note, in the absence of bouquet formation, centromeres have the ability to interact with the spindle pole body to mediate spindle assembly instead of telomeres, keeping chromosomes close to an interphase Rabl configuration [48]. It is possible that a more complex size-dependent pattern is propagated at the spindle pole body from transitioning between the Rabl configuration in interphase, the bouquet, and then centromere coupling.

Our findings from WT diploids and bouquet mutants guide us to update a previous coupling model [16], where centromeres are randomly paired to a revised model (Fig 6E) where bouquet formation would first help to establish chromosomal interactions based on chromosome size. The bouquet appears to serve as a chromosome size sorter, not only for homologous chromosomes as previously postulated [45] but also for non-homologous coupling. This sorting mechanism would rely on the degree of clustering forces and on the biophysical properties of chromosomes [45], as well as the overall chromosomal configuration away from telomeres. Specifically our results suggest the bouquet’s role in the mechanism for homolog pairing: this configuration sets up the chromosomes in a size-dependent alignment for coupling, as a first step to homolog recognition. As meiotically-programmed DSBs occur, and recombination-based homology searches begin, Zip1 becomes phosphorylated, releasing the couples [18], and repeated pairing partner switching ensues (speed-dating model) [16]. As chromosomes find their homologs, and begin to synapse, they are effectively removed from the coupling pool, incrementally restricting the possible couples. Longer chromosomes tend to become paired with their homologs earlier [15] and locked in through SC formation and recombination, whereas small chromosomes continue their non-homologous contacts. This late pairing phase is in concordance with data obtained on a smaller scale using electron microscopy [15].

While we found a preference for centromere coupling interactions based on chromosome size similarities, our data do not perfectly fit this pattern. Closer inspection of heatmaps reveals the presence of “cold” orthogonal diagonals, with non-homologous couples interacting less frequently. This brings the possibility that there are likely cold and hot spots for coupling interactions. In budding yeast, the 32 telomeres appear as 3–8 clusters in interphase [49, 50]. Could telomere clusters, present prior to the formation of the meiotic bouquet, play a role in establishing the interaction patterns observed in centromere coupling? We asked whether chromosomes found in the same telomere cluster are strong interacting partners in coupling. Telomere cluster assignments differed whether they were determined by genetics and chromosomal tagging methods [51], or derived from 4C genomic data [24, 52]. A coupling interaction pattern based on telomere clusters from Schober et al. [51] is highly significant in *spo11 ndj1* diploids (Randomization matrix test; p < 0.01), but not in *spo11*, *spo11 zip1*, or *spo11 rec8* diploid strains. Since *spo11 ndj1* yeast do not form the meiotic bouquet, preferential interactions based on prior telomere clusters might be favored during centromere coupling, compared to the chromosome size-dependent pattern observed in *spo11* and WT diploid yeast that go through the bouquet stage.

The role of centromere coupling remains unknown. It might be an initial step for chromosomes to query whether one chromosome is its homologous match, but since *zip1* (coupling and SC defective) mutants are capable of robust homolog pairing [53], coupling must be a redundant path for homolog pairing. Another function of coupling might be to block the deleterious establishment of recombination at centromeres of homologous chromosomes [17]. Centromeres likely constitute a special region for these interactions, providing a *cis* regulatory center for each chromosome where conditions must be met before SC formation is permitted. Although SC formation can initiate at sites other than the centromere, centromere synapsis generally occurs earlier [54]. Indeed Zip1 has been shown to deflect deleterious crossing over in the immediate centromere vicinity [55].

Identification of additional functional requirements for centromere coupling will likely provide more clues into its role in early meiosis. In this study, we have proven the advantages of genomics approaches to characterize a biological phenomenon. While more technically challenging, expansive and time consuming than standard methods, only such a strategy would have been able to identify pairwise trends systematically with this higher level of confidence.

## Materials and Methods

### Strains

Yeast strains are isogenic with BR1919-8B (S1 Table) [56].

### Sporulation

Strain growth was performed as described [39]. Cultures were grown first for 24 h in YPADU at 30°C to early stationary phase. Then cultures were resuspended in 2% sporulation media (2% potassium acetate) to a final cell concentration of 2–4 X 10^7^ cells/mL, as determined by OD_600_ on a spectrophotometer. About 4 X 10^9^ cells were needed per 3C sample [35]. 200 mL cultures were grown in 2 L flasks to promote good sporulation by providing adequate oxygenation (6 L flasks for 5-time points in WT diploid cells). Haploid cells were grown for 20 h in sporulation media while diploid cells were grown for 14 h.

### Chromosome spreading and immunofluorescence microscopy

Meiotic chromosome spreading was performed as previously described [39]. Staining was performed using antibodies against Red1[57] and against the Myc epitope (9E10) to detect Ctf19-Myc [16]. Cy3-conjugated anti-mouse (Ctf19) and FITC-conjugated anti-rabbit (Red1) secondary antibodies were used for detection. Slides were also stained with DAPI in the mounting media to observe compact spreading and core formation. Meiotic chromosome spreads were visualized on a Nikon E800 microscope and images were visualized on the IPLab software, as previously described [58]. Immunofluorescence was performed on WT diploids at various times throughout meiosis to determine centromere organization (Ctf19) and the appearance of SC components (Zip1 and Red1).

For the tetO-tetR-mCherry/lacO-lacI-GFP experiment, primary antibodies against c-MYC (9E10 mouse), GFP (chicken) and m-Cherry (rabbit) were visualized with the following secondary antibodies: anti-mouse CY5, anti-chicken FITC and anti-rabbit Texas Red. Nuclear spreads were screened by DAPI for compact spreading and core formation. Among those spreads, the kinetochore arrangement (Ctf19-Myc staining) was scored for phase and spreading. Early spreads contain a single focus of kinetochores or a loosened bundle and are not useful for determining coupling. Other spreads may contain greater than 8 Ctf19 foci and represent disruption of coupling from physical spreading. Only those nuclear spreads that met criteria for being coupled (~8 Ctf foci) were scored for pairing. Three independent biological replicates were carried out for each strain and fifty nuclei were scored for each experiment.

### Chromosome conformation capture double digestion (3C2D)

The 3C procedure followed previously described protocols from the Dekker lab with few modifications [25, 35, 36]. An extra chloroform extraction step and a post-precipitation wash with 70% ethanol were added for increased purity of 3C libraries. For the double digestion, EcoRI-HF and MfeI-HF (NEB) generated cohesive ends that were compatible, yet recognizing slightly different sequences (GAATTC and CAATTG respectively). They were selected due to their extremely low star activity, extended enzymatic half-life suitable for overnight digestion and stronger activity in samples of lower purity, making them appropriate for digestion of crosslinked chromatin. 50 U of each enzyme were used per tube (each sample is divided into 40 individual tubes at this step; see [35]).

For quality control, we ensured that formaldehyde crosslinking enabled preferential ligations of fragments in close proximity in the nucleus [25]. On our 3C2D libraries, we amplified fragments with primer pairs located 10 kb and 80 kb away on chromosome 8. We used a control representing randomly-ligated, not crosslinked genomic DNA, in which proximal and distal fragments are present in similar abundance [25]. Several dilutions of templates were used for PCR amplification. PCR products were detected by gel electrophoresis on a 2% agarose gel. Band intensity was determined using ImageJ (NIH). 3C2D libraries that did not show a formaldehyde-mediated enrichment for proximal intra-chromosomal interactions over distal ones were discarded.

### Primer design and Taqman probe design

Primers were designed 100–150 bp away from the closest *CEN*-proximal MfeI or EcoRI site on each side of the *CEN*, with length between 18 and 24 bp optimally and a melting temperature around 56–58°C (S2 Table). Each primer is oriented towards the restriction site in order to amplify fragments from different chromosomes ligated together. Taqman probes were designed as recommended for mammalian 3C-qPCR [32] on the opposite strand from the primer and around 10–60 bp away from the restriction site. Taqman 5’FAM-3’TAMRA probes (QuickProbes, Operon) were designed for annealing at 65–68°C, with the following constraints: 20–36 bp in length, more Cs than Gs, no G at the 5’ end to prevent quenching, no stretch of 4 identical nucleotides and no more than 2 C’s and/or G’s in the last 5 nucleotides of the 3’ end (S3 Table).

### Real-time quantitative PCR (qPCR) analysis of 3C2D samples (3C2D-qPCR)

All 480 possible interactions (*CEN1* left vs. *CEN2* left, *CEN1* left vs. *CEN2* right, *CEN1* left vs. *CEN3* left, …, *CEN1* right vs. *CEN2* left, *CEN1* right vs. *CEN2* right, …) were analyzed by performing qPCR reactions in triplicates and diluted 2-, 4- and 8-fold. Individual reactions were set up following previously established guidelines [32]: 2 μL of each primer (2.5 μM), 1 μL Taqman probe (1.5 μM), 5 μL QuantiTect Probe PCR master mix (Qiagen), and 1 μL diluted 3C2D or control sample. qPCR reactions were run on a LightCycler 480 (Roche) with the following parameters: 1) enzyme activation for 15 min. at 95°C, and 2) 55 cycles of amplification with a 15 s denaturation at 95°C, a 60 s amplification at 60°C and a single fluorescence acquisition.

The “Second derivative maximum” analytical tool in the LightCycler480 was used to obtain Crossing point values (C_p_). For each individual reaction, the amplification curve was visually inspected to ensure that the reported C_p_ value was plausible for the exponential phase of the curve. C_p_ values were adjusted according to the sample dilution in individual qPCR reactions, including a correction for successful completion when certain dilutions failed to amplify. Values were averaged for each of 120 combinations (*CEN1* vs. *CEN2*, *CEN1* vs. *CEN3*, etc.) to generate a raw interaction frequency matrix. Each combination is formed by the following interactions: *CENX* Left-*CENY* Left, *CENX* Left-*CENY* Right, *CENX* Right-*CENY* Left, and *CENX* Right-*CENY* Right. The resulting interaction frequencies were normalized as described in the analytical section of the original 3C-qPCR protocol [32]. First, they were normalized to the amplification efficiencies obtained from control samples (randomly-ligated genomic DNA). Second, they were normalized according to the DNA concentration of the 3C2D library (internal loading control). In this case, we performed RT-qPCR with the SYBR Green system on a LightCycler480 (Roche) to quantify an internal product amplified from a primer pair which does not amplify across MfeI or EcoRI sites, using dilutions of the 3C2D library (1:12.5, 1:25, 1:50, 1:100, 1:200) [32]. Finally, since we are looking only at inter-chromosomal interactions between non-homologous centromeres, our experimental design protects from artifactual peaks that can arise when local conformation of chromatin influences the detection of intra-chromosomal interactions [38].

### Data analysis

For individual genotypes, heat maps were generated in R using interaction frequencies calculated as described above. We used the color scheme “YlOrRd” from package RColorBrewer. Darker shades of red indicate a higher level of interaction. For visualization purposes, we separated chromosomes in three groups based on chromosome size, using k-means clustering. For the multiple time points in a WT diploid, differences of normalized interaction frequencies were plotted on a heatmap to compare their relative progression (early→mid, mid→late, and early→late). In this case, heatmaps were unscaled and generated using the color scheme “RdBu”, with white meaning no changes, red for increases, and blue for decreases.

To test the significance of the pairwise pattern based on similarities of chromosome lengths, we used a non-parametric testing procedure. For each chromosome (out of 16), we identify the number of times one of three closest chromosomes in length to that particular chromosome would be also amongst the three chromosomes with the highest interaction frequencies with that particular chromosome. We summed the number of times we had such a case across all 16 chromosome. We next simulated the null distribution by randomizing the matrix of interaction frequencies, then selecting the three strongest interacting partners (out of 15) for each particular chromosome and asking whether they would be one of the three closest chromosomes in length as well. We repeated this 15 more times (16 total) and summed to obtain the grand total of top 3 interactions with top 3 chromosomes closest in size across all 16 chromosomes. This constitutes one iteration. We performed this procedure 100,000 times. The p-value is given by the fraction of random iterations with greater or equal association between IF strength and chromosome size similarities (grand total) than found experimentally for each genotype. A similar randomization approach was used on a subset of the matrix when comparing the four chromosomes of shortest size, and the four chromosomes of largest size.

For arm homology, a similar non-parametric procedure was performed, except that we used, for each chromosome, the three chromosomes with the highest level of arm homology as determined from the figures and from the raw data of ORF homology [40].

For the agreement between haploid and diploid *spo11* strains at the top of the interaction list, we used a similar non-parametric strategy, except that, for each chromosome, we randomly selected 5 chromosomes for the haploid strain and 5 chromosomes for the diploid strain, asking how many chromosomes overlap. Then we summed across all 16 chromosomes and performed 100,000 iterations.

Data to generate all heatmaps and graphs are available from the Dryad Digital Repository: http://dx.doi.org/10.5061/dryad.71425. Additional data and a few sample codes are deposited in GitHub: https://github.com/plefrancois/CENcoupling.

## Supporting Information

S1 Fig Size distribution of restriction fragments encompassing the centromere generated by standard single digestion 3C and by double-digestion 3C (3C2D). The number of centromeric fragments (out of 16) and their size in bins of 2 kilobases (kb) are plotted for a single EcoRI digestion (blue), for a single MfeI digestion (red), and for a combined EcoRI-MfeI digestion (black).
(TIF)

S2 Fig **Average number of qPCR cycles for all possible 480 interactions using the same concentration of control DNA template from haploid (A) and diploid (B) strains.** Primer pairs were assessed by Taqman qPCR assay on control libraries consisting of randomly-ligated, non-crosslinked genomic DNA representing all possible fragments in equimolar ratios. Dotted blue lines indicate the median values.
(TIF)

S3 Fig Absolute differences in the average number of qPCR cycles for the same Taqman qPCR reaction between haploid and diploid control libraries. The dotted blue lines indicate the median difference (0.61 cycle).
(TIF)

S4 Fig Amplification of intra-chromosomal restriction fragments as a quality control for 3C2D libraries. Crosslinking enhances ligation of proximal fragments compared to distal fragments. **(A)** Design of intra-chromosomal primers on chromosome 8. Using a constant primer (black arrow), amplification was carried on with primers located 10 kb away (proximal; red arrow) or 80 kb away (distal; blue arrow). **(B)** Top: Detection of PCR products by gel electrophoresis on a 2% agarose gel. Concentrations (in ng/μL) of serially-diluted libraries are given above all lanes. Bottom: Quantification of band intensities from above gels for primer pairs located 10 kb away (red) and 80 kb away (blue) on chromosome 8. Band intensities (in arbitrary units) were obtained using ImageJ software and plotted according to the concentration of the library dilution. Left: The DNA template of the PCR reactions is the control library consisting of non-crosslinked, randomly-ligated genomic DNA. Right: The DNA template of the reactions is the 3C2D experimental sample from digested, crosslinked chromatin ligated under dilute conditions to favor linkage of fragments crosslinked together.
(TIF)

S5 Fig Heatmap of ranked interaction frequencies between non-homologous centromeres in *spo11* diploids. Centromeres are arranged from left to right and bottom to top according to their respective chromosome length, from shortest to longest. For each centromere, darker shades of red indicate a rank closer to 1 for that interaction (strongest).
(TIF)

S6 Fig Heatmap of ranked interaction frequencies between non-homologous centromeres in *spo11 zip1* diploids. Centromeres are arranged from left to right and bottom to top according to their respective chromosome length, from shortest to longest. For each centromere, darker shades of red indicate a rank closer to 1 for that interaction (strongest).
(TIF)

S7 Fig Heatmap of differences in raw interaction frequencies between *spo11* and *spo11 zip1* diploids. Centromeres are arranged from left to right and bottom to top according to their respective chromosome length, from shortest to longest. Heatmaps were unscaled, with white meaning no changes, red for increases, and blue for decreases. Please note the log2 scale on the color key for interaction frequencies. S7 Fig needs to be interpreted in light of Fig 2, as differences could arise from the different ranges of interaction values within the two genotypes, including some couples with barely detectable amplification in *spo11 zip1*, which can cause a low interaction to become aberrantly high in comparison.
(TIF)

S8 Fig Heatmap of ranked interaction frequencies between non-homologous centromeres in *spo11* haploids. Centromeres are arranged from left to right and bottom to top according to their respective chromosome length, from shortest to longest. For each centromere, darker shades of red indicate a rank closer to 1 for that interaction (strongest).
(TIF)

S9 Fig Heatmap of ranked interaction frequencies between non-homologous centromeres in *spo11 zip1* haploids. Centromeres are arranged from left to right and bottom to top according to their respective chromosome length, from shortest to longest. For each centromere, darker shades of red indicate a rank closer to 1 for that interaction (strongest).
(TIF)

S10 Fig Heatmap of differences in raw interaction frequencies between *spo11* and *spo11 zip1* haploids. Centromeres are arranged from left to right and bottom to top according to their respective chromosome length, from shortest to longest. Heatmaps were unscaled, with white meaning no changes, red for increases, and blue for decreases. Please note the log2 scale on the color key for interaction frequencies. S10 Fig needs to be interpreted in light of Fig 3, as differences could arise from the different ranges of interaction values within the two genotypes, including some couples with barely detectable amplification in *spo11 zip1*, which can cause a low interaction to become aberrantly high in comparison.
(TIF)

S11 Fig Meiotic progression in a wild-type diploid strain. At each time point after meiotic induction (initiation of sporulation), an aliquot of the master culture was taken to determine meiotic progression from centromere organization (Ctf19) and appearance of SC components (Zip1 and Red1) in WT diploids by chromosome spreading. Spreads were classified as clustered centromeres (2–4 large foci; plain bars), separated/coupled centromeres (~16 Ctf foci; dotted bars), presence of SC (at least one linear stretch of Zip1/Red1; lined bars), and late MI/early MII (grey bars). About 50 individual spreads were assessed per independent replicate per time point. The percentages of spreads in the four categories are given on the y-axis (mean +/- standard deviation), for each time point (8h, 9h, 10h, 11h and 14h).
(TIF)

S12 Fig Heatmaps from meiotic time points after meiotic induction (initiation of sporulation) in a wild-type diploid strain. **(A)** Heatmap of normalized interaction values between non-homologous centromeres at each time point (8h, 9h, 10h, 11h and 14h). Centromeres are arranged from left to right and bottom to top according to their respective chromosome length, from shortest to longest. Darker shades of red indicate a higher level of interaction between non-homologous centromeres. Please note the log2 scale on the color key for interaction frequencies. **(B)** Heatmaps of ranked interaction frequencies between non-homologous centromeres at each time point (8h, 9h, 10h, 11h and 14h). Centromeres are arranged from left to right and bottom to top according to their respective chromosome length, from shortest to longest. For each centromere, darker shades of red indicate a rank closer to 1 for that interaction (strongest).
(TIF)

S13 Fig Status of centromeres (coupled/separated vs. clustered) of various *spo11* yeast strains at the time of cell harvesting. An aliquot of the cultures used for 3C2D-qPCR was taken to determine the centromere organization (Ctf19) by chromosome spreading. Spreads were classified as either separated/coupled centromeres (lined bars), or clustered centromeres/other status (plain bars), similarly to previous reports [17, 44]. About 50 individual spreads were assessed per independent replicate. The percentages of spreads in the two categories are given on the y-axis (mean +/- standard deviation), for multiple haploid and diploid yeast strains of various genotypes.
(TIF)

S14 Fig Heatmap of ranked interaction frequencies between non-homologous centromeres in *spo11 ndj1* diploids. Centromeres are arranged from left to right and bottom to top according to their respective chromosome length, from shortest to longest. For each centromere, darker shades of red indicate a rank closer to 1 for that interaction (strongest).
(TIF)

S15 Fig Heatmap of ranked interaction frequencies between non-homologous centromeres in *spo11 rec8* diploids. Centromeres are arranged from left to right and bottom to top according to their respective chromosome length, from shortest to longest. For each centromere, darker shades of red indicate a rank closer to 1 for that interaction (strongest).
(TIF)

S1 Table Yeast strains used in this study. (DOC)

S2 Table Primer sequences used for 3C2D-qPCR. (DOC)

S3 Table Taqman probes used for 3C2D-qPCR. (DOC)
