# Supplementary figures and images for: Multiple Pairwise Analysis of Non-homologous Centromere Coupling Reveals Preferential Chromosome Size-Dependent Interactions and a Role for Bouquet Formation in Establishing the Interaction Pattern
Source: PLoS Genet. 2016 Oct 21;12(10):e1006347. doi: 10.1371/journal.pgen.1006347 (PMC5074576; doi:10.1371/journal.pgen.1006347)

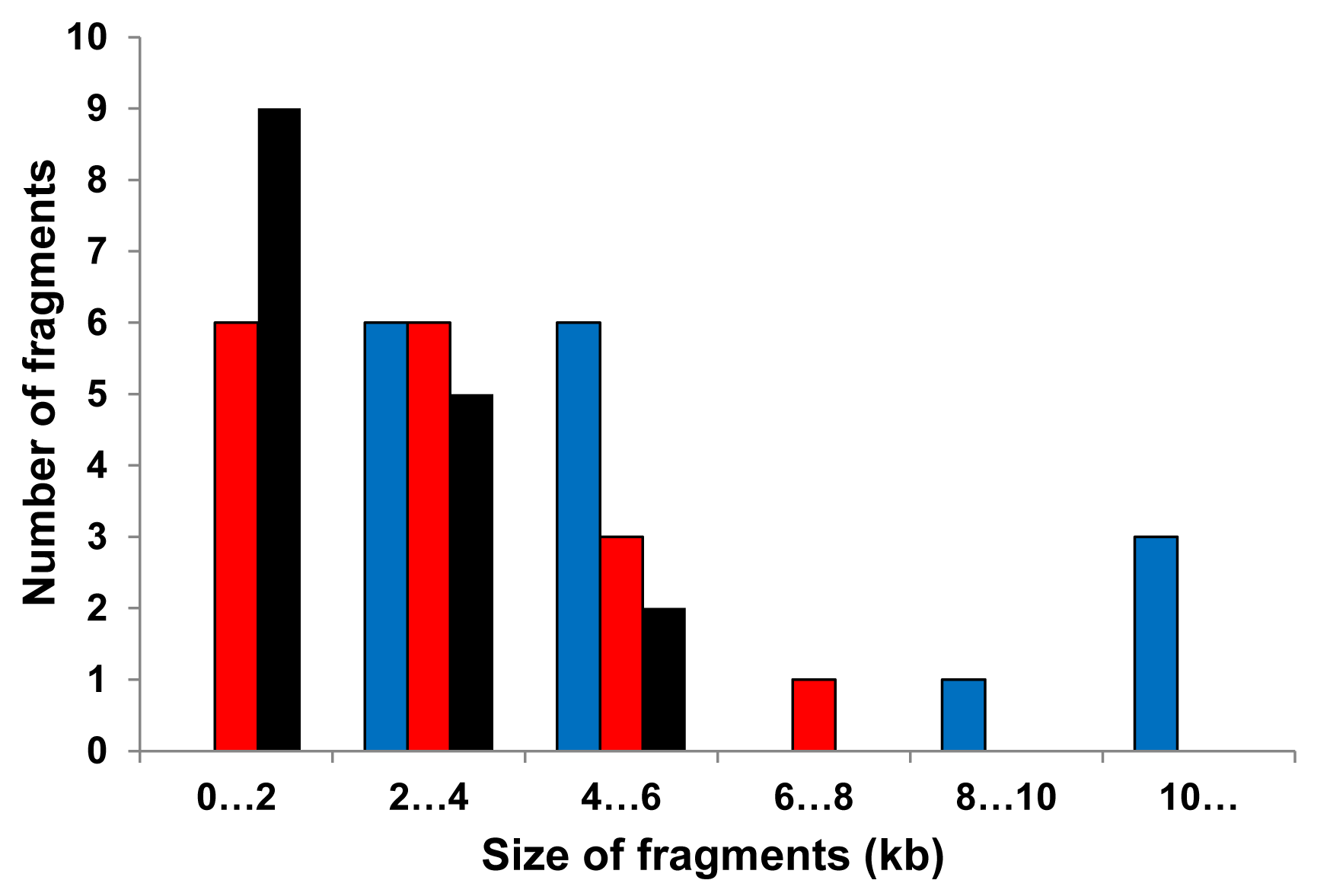

Supplement: S1 Fig — The number of centromeric fragments (out of 16) and their size in bins of 2 kilobases (kb) are plotted for a single EcoRI digestion (blue), for a single MfeI digestion (red), and for a combined EcoRI-MfeI digestion (black). (TIF) [file pgen.1006347.s001.tif]

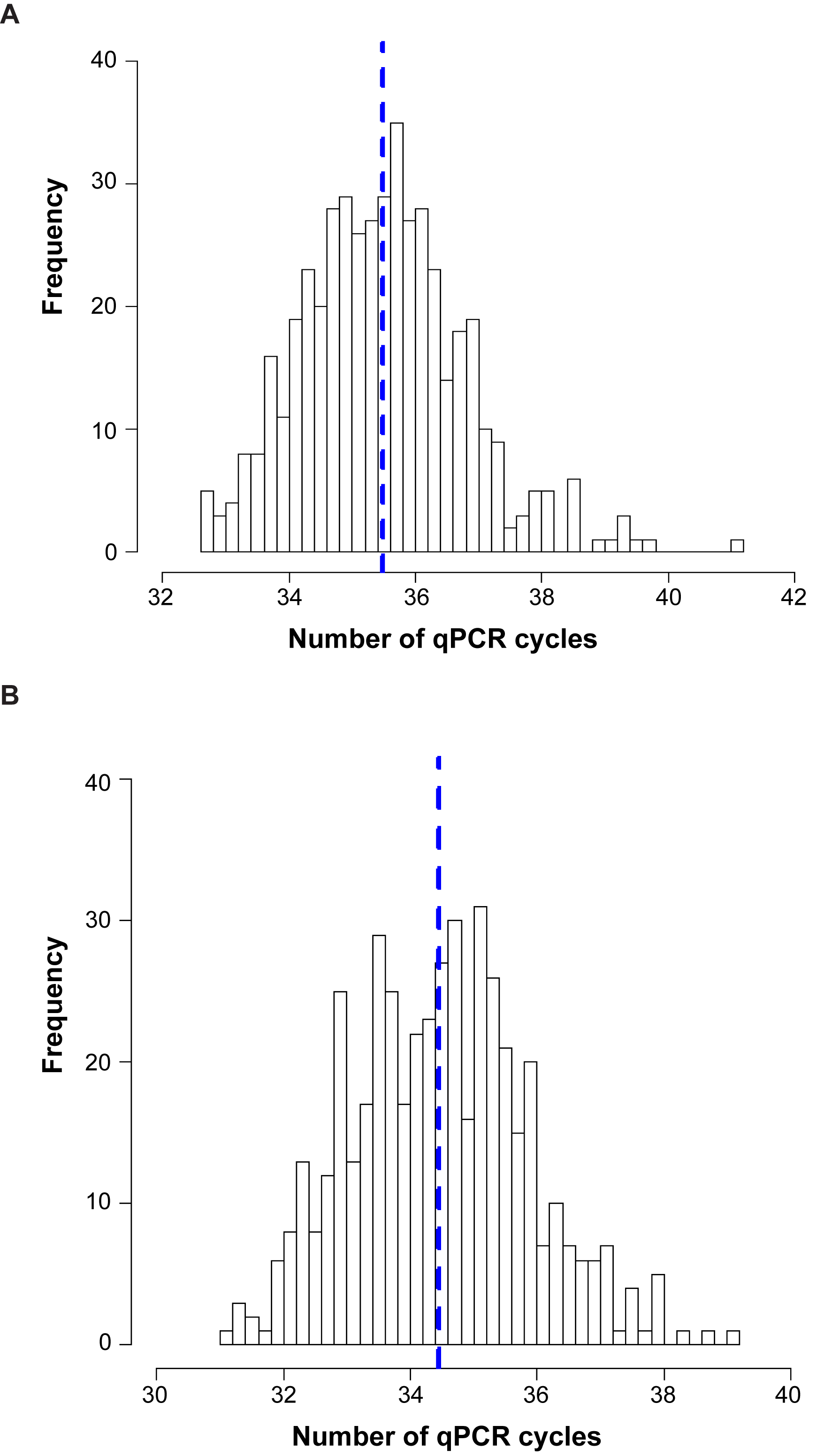

Supplement: S2 Fig — Average number of qPCR cycles for all possible 480 interactions using the same concentration of control DNA template from haploid (A) and diploid (B) strains. Primer pairs were assessed by Taqman qPCR assay on control libraries consisting of randomly-ligated, non-crosslinked genomic DNA representing all possible fragments in equimolar ratios. Dotted blue lines indicate the median values. (TIF) [file pgen.1006347.s002.tif]

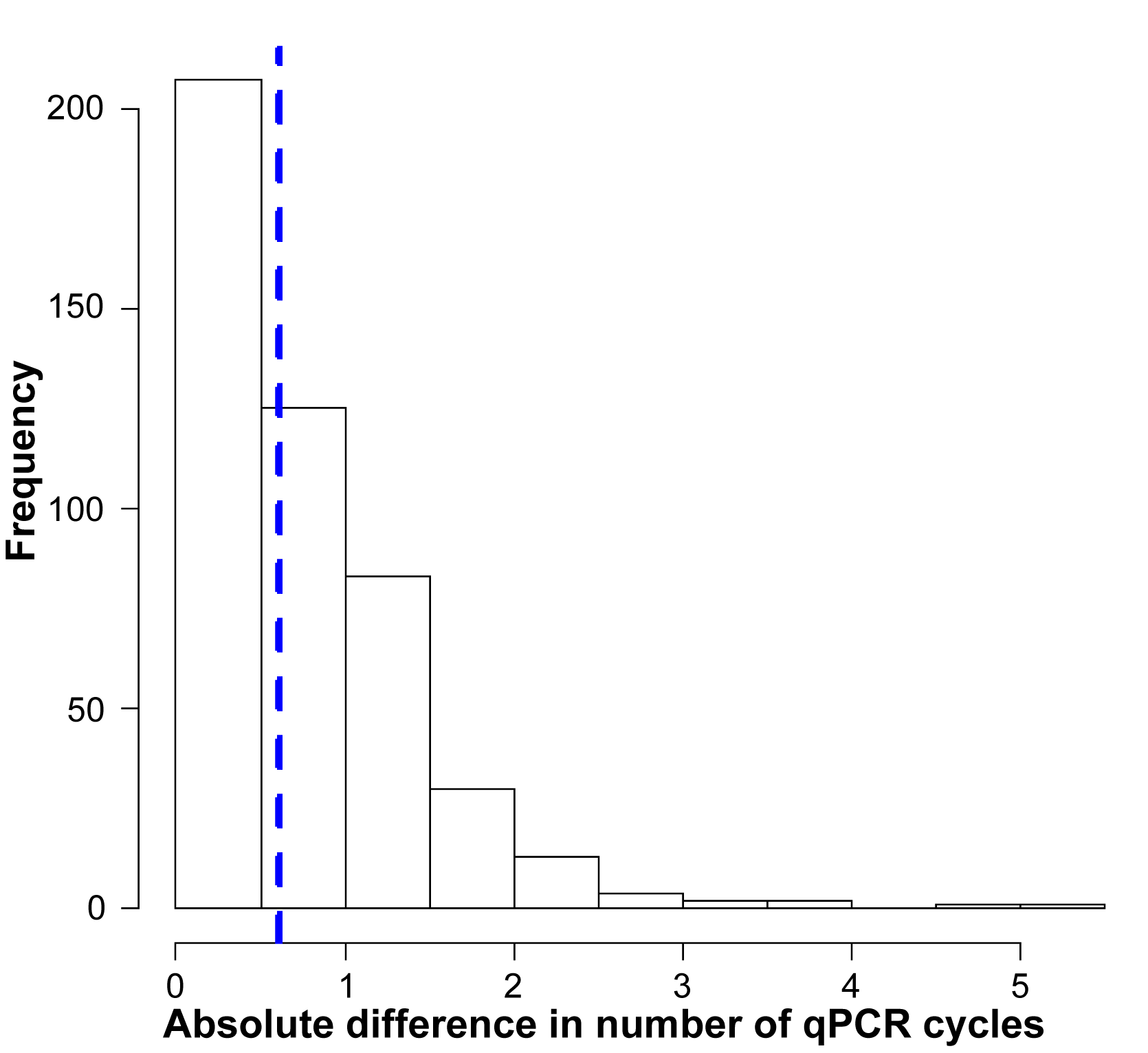

Supplement: S3 Fig — The dotted blue lines indicate the median difference (0.61 cycle). (TIF) [file pgen.1006347.s003.tif]

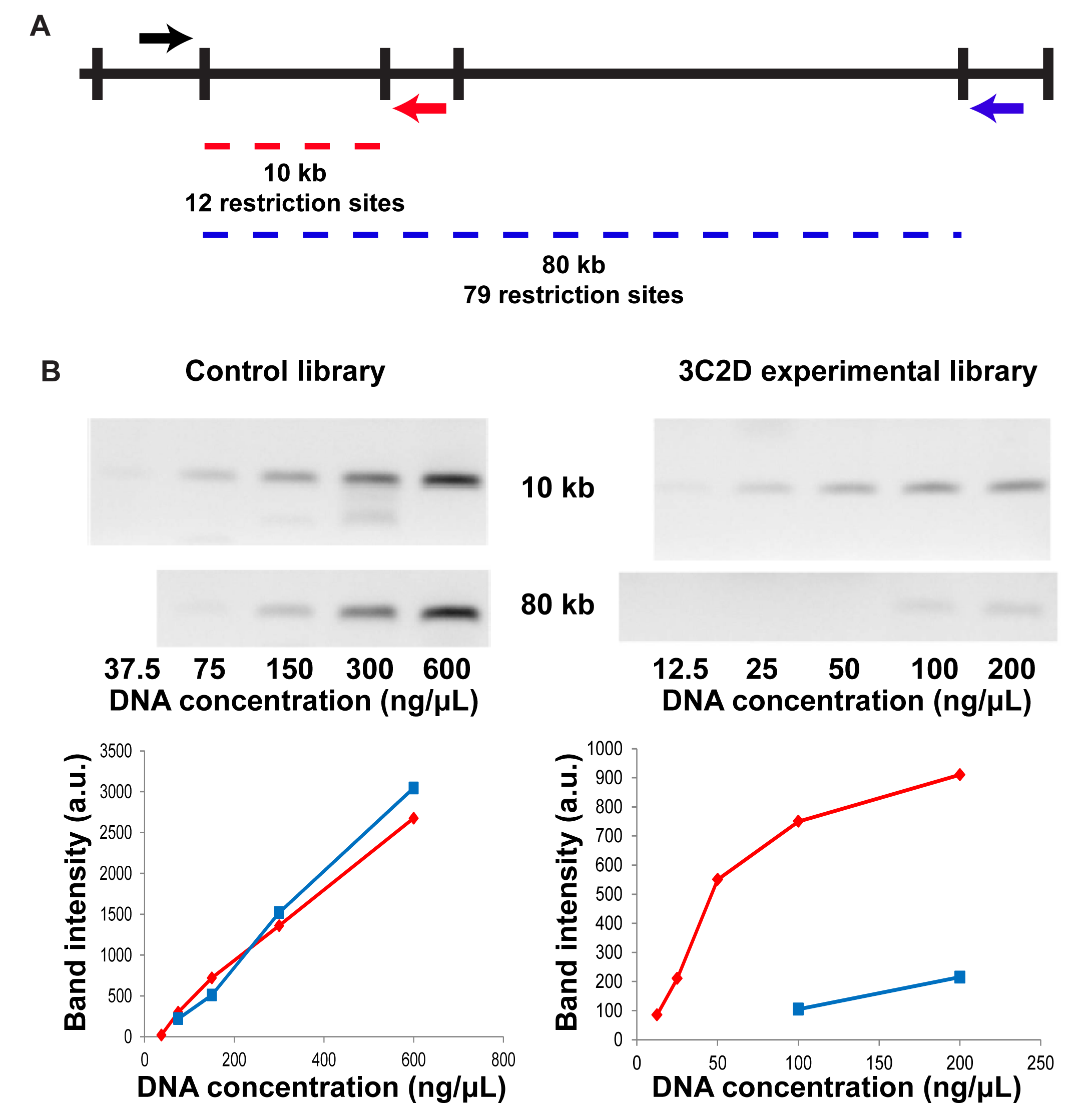

Supplement: S4 Fig — Crosslinking enhances ligation of proximal fragments compared to distal fragments. (A) Design of intra-chromosomal primers on chromosome 8. Using a constant primer (black arrow), amplification was carried on with primers located 10 kb away (proximal; red arrow) or 80 kb away (distal; blue arrow). (B) Top: Detection of PCR products by gel electrophoresis on a 2% agarose gel. Concentrations (in ng/μL) of serially-diluted libraries are given above all lanes. Bottom: Quantification of band intensities from above gels for primer pairs located 10 kb away (red) and 80 kb away (blue) on chromosome 8. Band intensities (in arbitrary units) were obtained using ImageJ software and plotted according to the concentration of the library dilution. Left: The DNA template of the PCR reactions is the control library consisting of non-crosslinked, randomly-ligated genomic DNA. Right: The DNA template of the reactions is the 3C2D experimental sample from digested, crosslinked chromatin ligated under dilute conditions to favor linkage of fragments crosslinked together. (TIF) [file pgen.1006347.s004.tif]

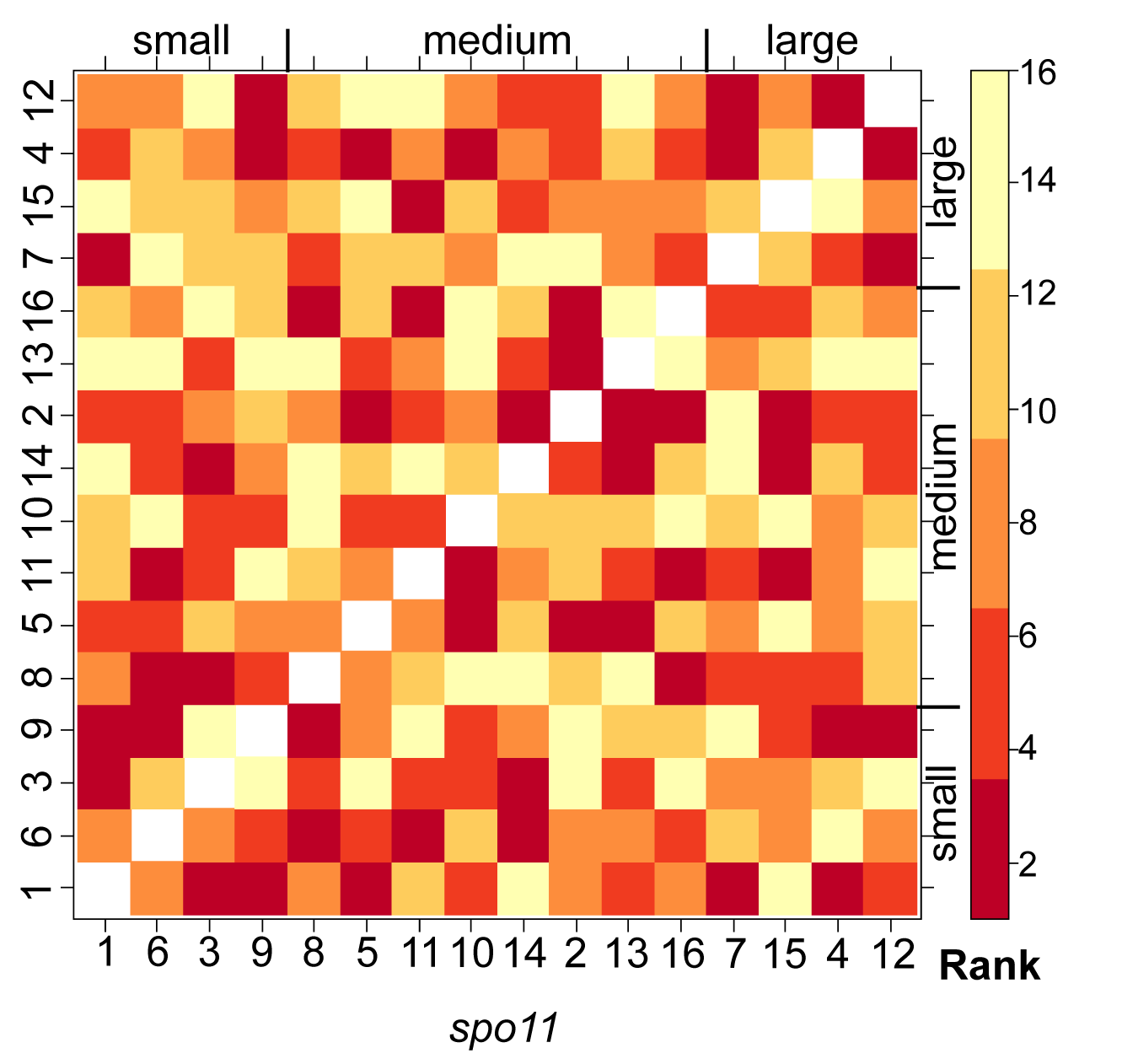

Supplement: S5 Fig — Centromeres are arranged from left to right and bottom to top according to their respective chromosome length, from shortest to longest. For each centromere, darker shades of red indicate a rank closer to 1 for that interaction (strongest). (TIF) [file pgen.1006347.s005.tif]

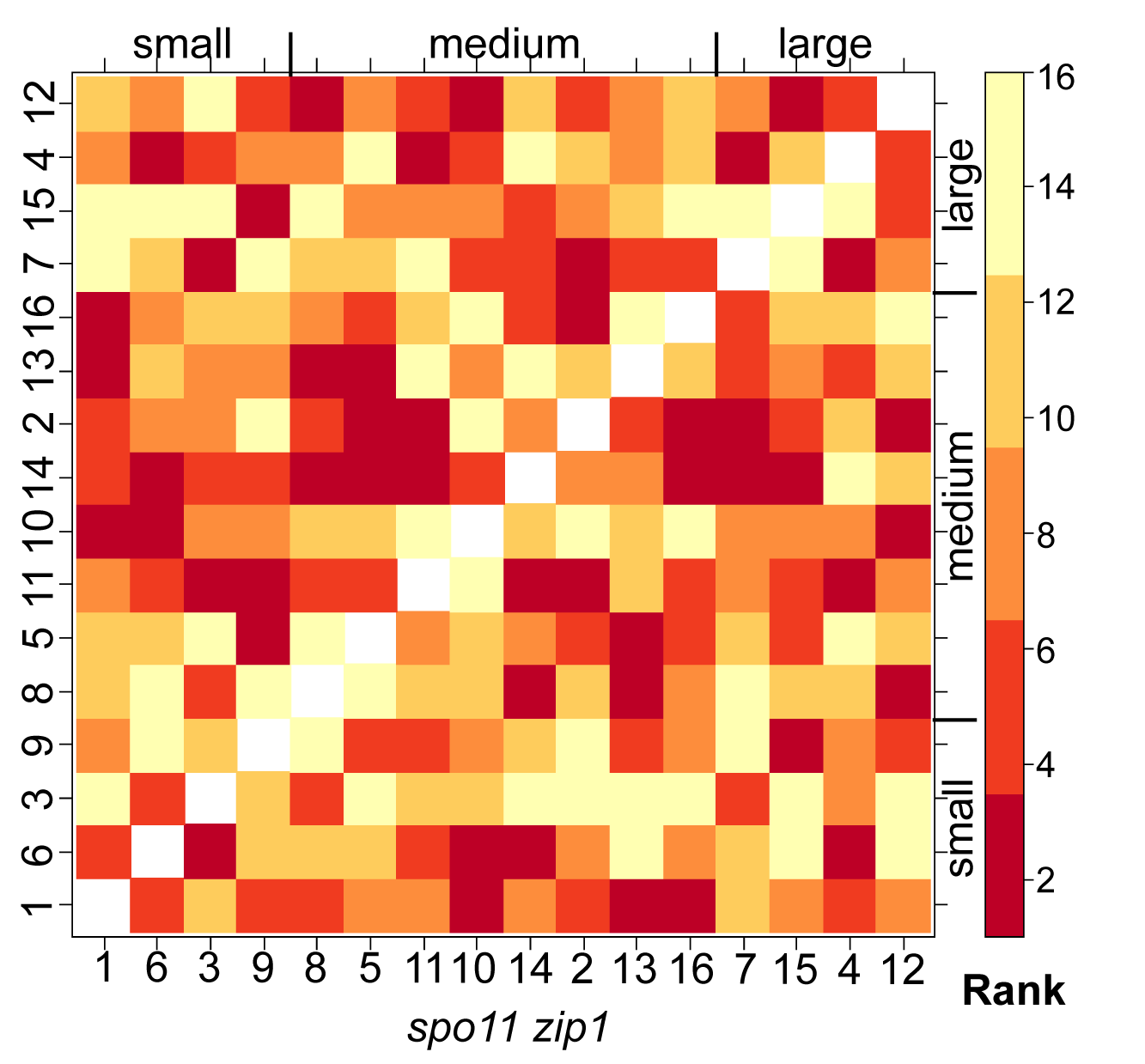

Supplement: S6 Fig — Centromeres are arranged from left to right and bottom to top according to their respective chromosome length, from shortest to longest. For each centromere, darker shades of red indicate a rank closer to 1 for that interaction (strongest). (TIF) [file pgen.1006347.s006.tif]

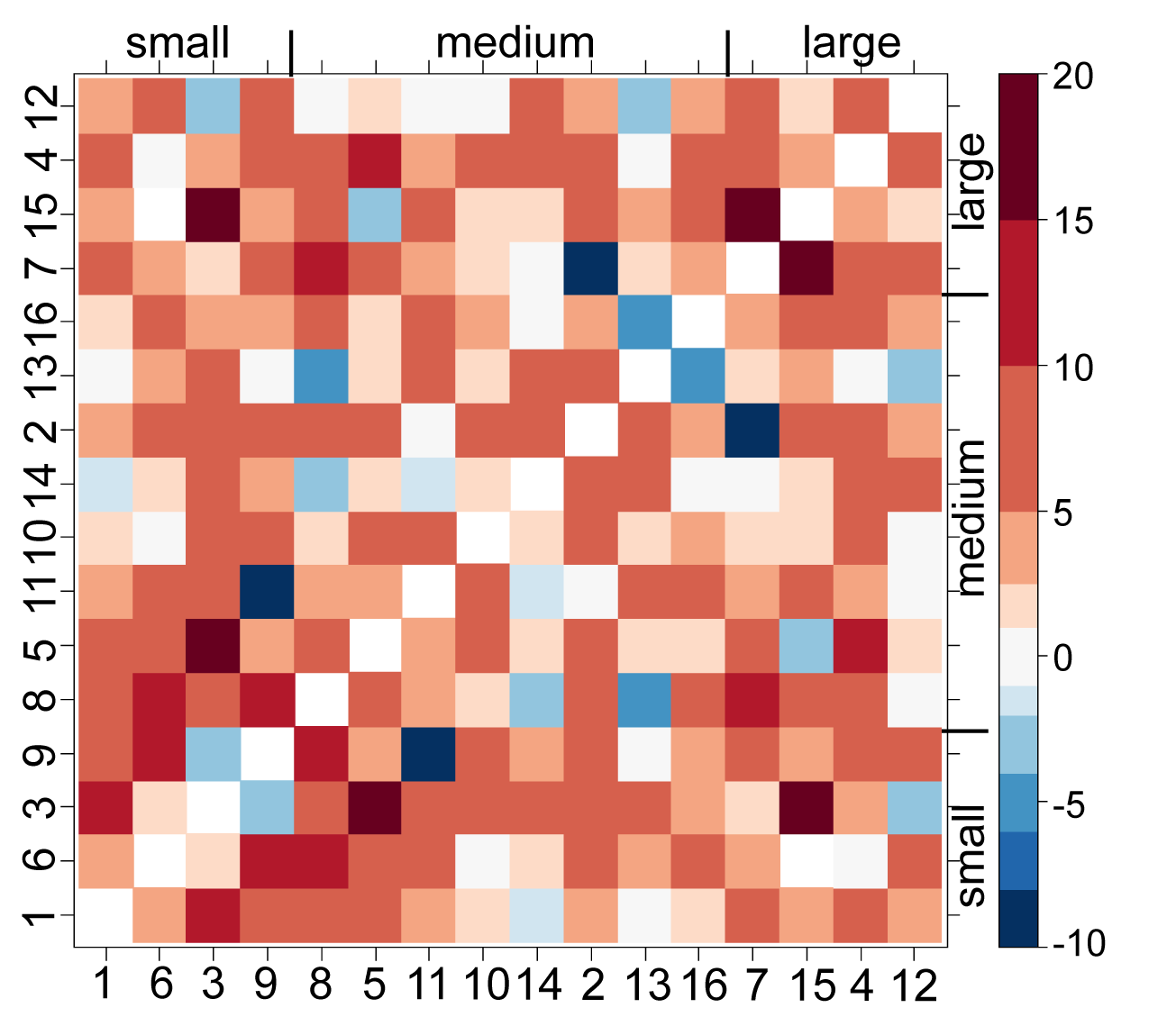

Supplement: S7 Fig — Centromeres are arranged from left to right and bottom to top according to their respective chromosome length, from shortest to longest. Heatmaps were unscaled, with white meaning no changes, red for increases, and blue for decreases. Please note the log2 scale on the color key for interaction frequencies. S7 Fig needs to be interpreted in light of Fig 2, as differences could arise from the different ranges of interaction values within the two genotypes, including some couples with barely detectable amplification in spo11 zip1, which can cause a low interaction to become aberrantly high in comparison. (TIF) [file pgen.1006347.s007.tif]

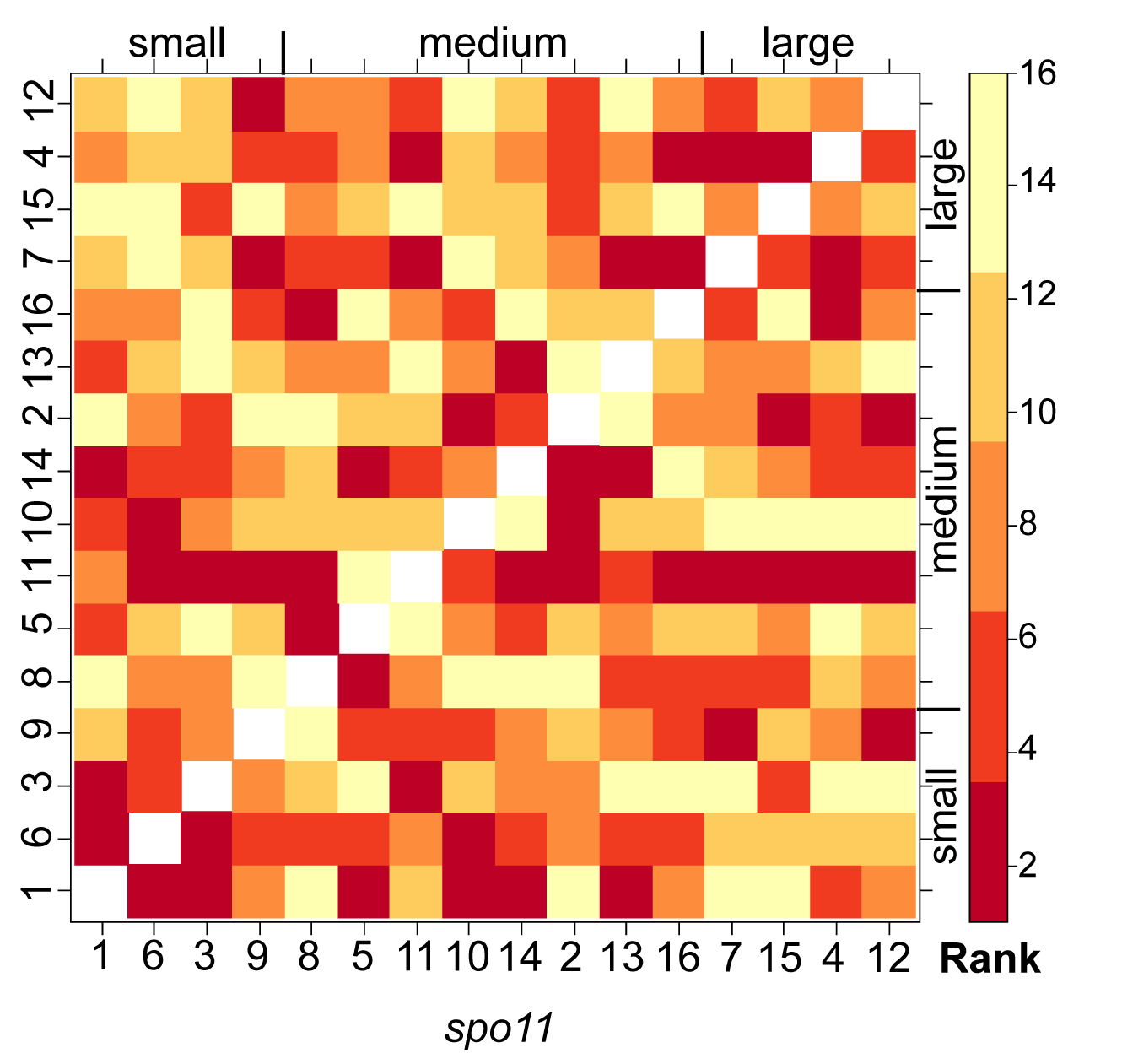

Supplement: S8 Fig — Centromeres are arranged from left to right and bottom to top according to their respective chromosome length, from shortest to longest. For each centromere, darker shades of red indicate a rank closer to 1 for that interaction (strongest). (TIF) [file pgen.1006347.s008.tif]

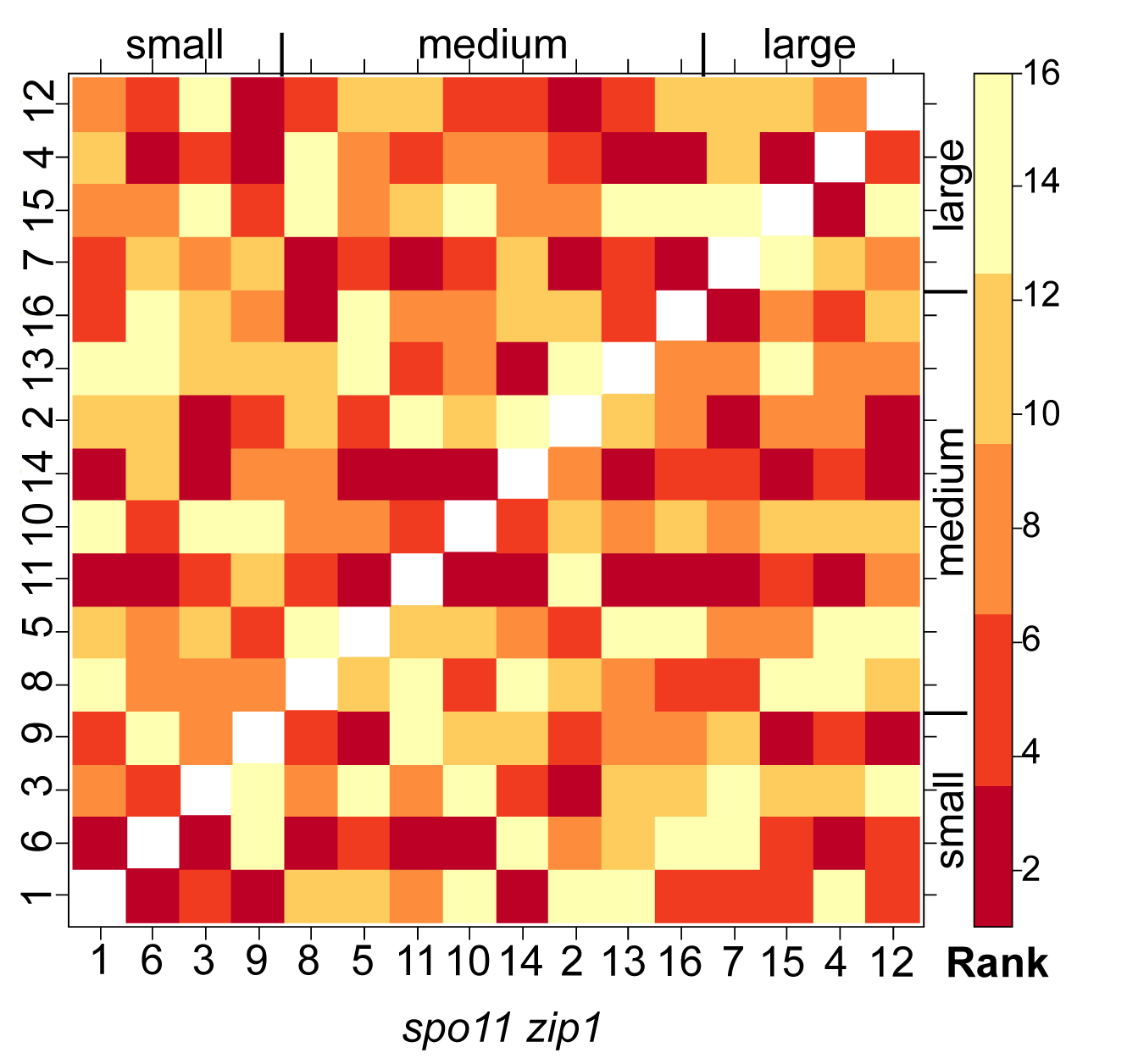

Supplement: S9 Fig — Centromeres are arranged from left to right and bottom to top according to their respective chromosome length, from shortest to longest. For each centromere, darker shades of red indicate a rank closer to 1 for that interaction (strongest). (TIF) [file pgen.1006347.s009.tif]

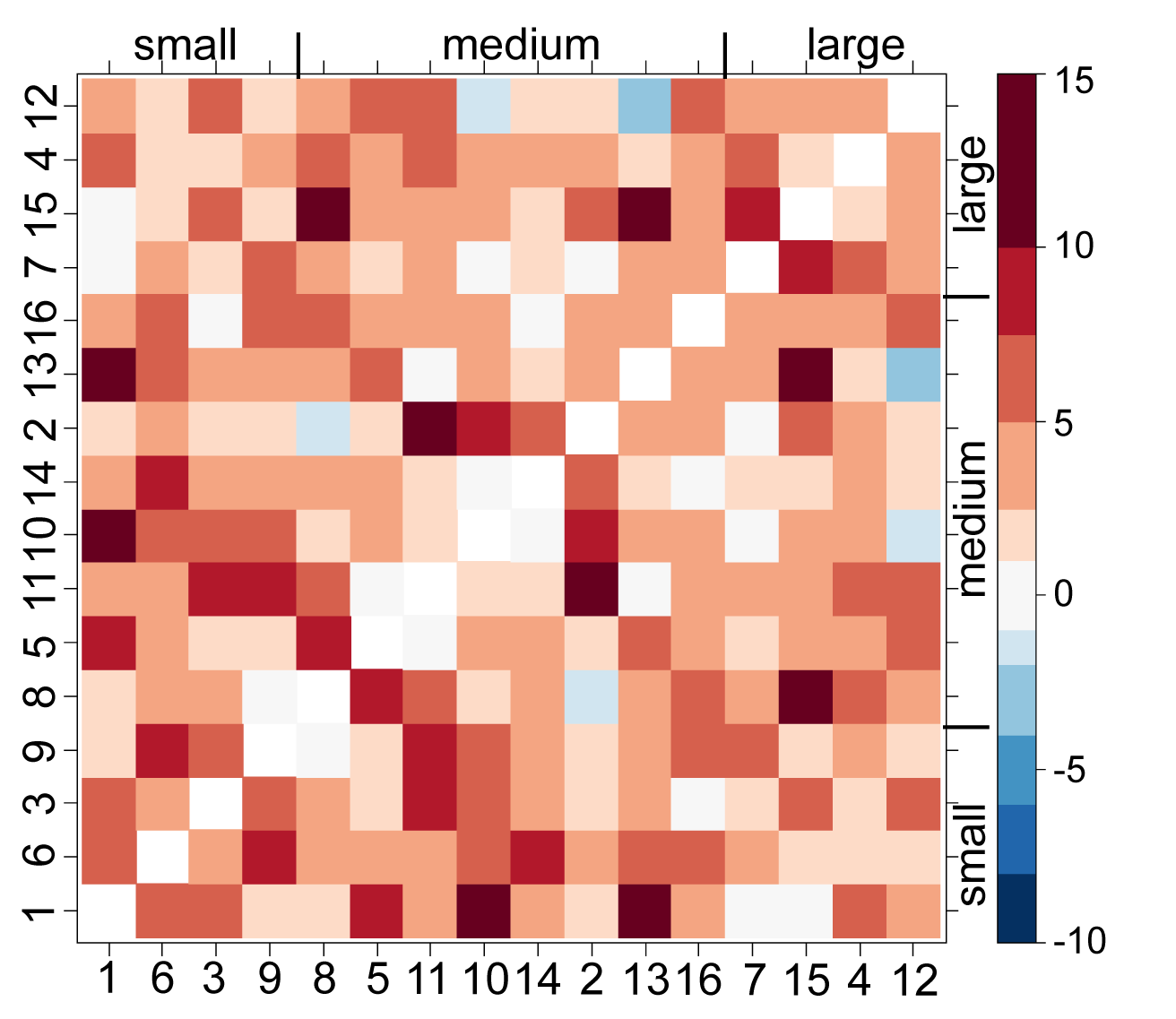

Supplement: S10 Fig — Centromeres are arranged from left to right and bottom to top according to their respective chromosome length, from shortest to longest. Heatmaps were unscaled, with white meaning no changes, red for increases, and blue for decreases. Please note the log2 scale on the color key for interaction frequencies. S10 Fig needs to be interpreted in light of Fig 3, as differences could arise from the different ranges of interaction values within the two genotypes, including some couples with barely detectable amplification in spo11 zip1, which can cause a low interaction to become aberrantly high in comparison. (TIF) [file pgen.1006347.s010.tif]

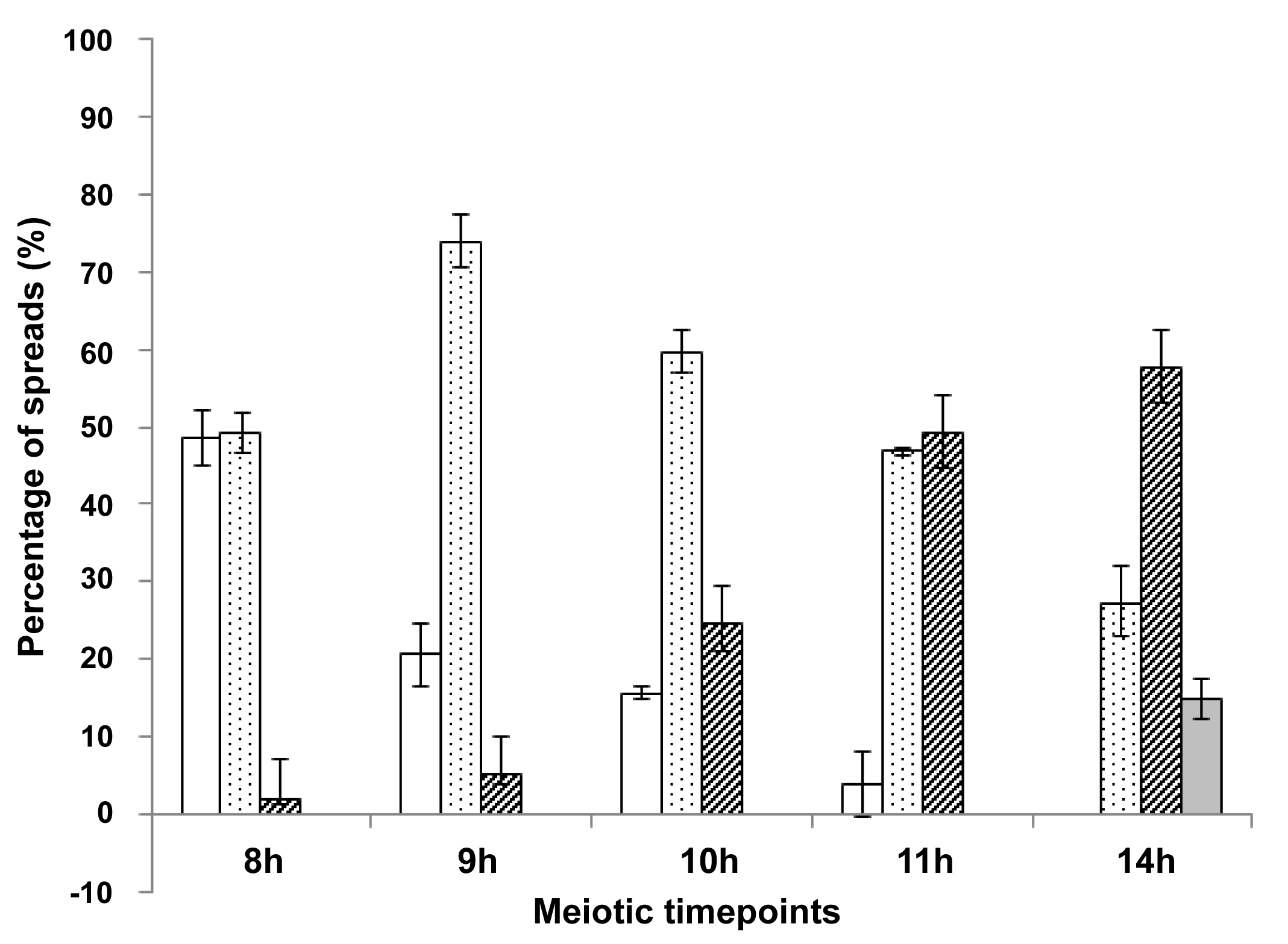

Supplement: S11 Fig — At each time point after meiotic induction (initiation of sporulation), an aliquot of the master culture was taken to determine meiotic progression from centromere organization (Ctf19) and appearance of SC components (Zip1 and Red1) in WT diploids by chromosome spreading. Spreads were classified as clustered centromeres (2–4 large foci; plain bars), separated/coupled centromeres (~16 Ctf foci; dotted bars), presence of SC (at least one linear stretch of Zip1/Red1; lined bars), and late MI/early MII (grey bars). About 50 individual spreads were assessed per independent replicate per time point. The percentages of spreads in the four categories are given on the y-axis (mean +/- standard deviation), for each time point (8h, 9h, 10h, 11h and 14h). (TIF) [file pgen.1006347.s011.tif]

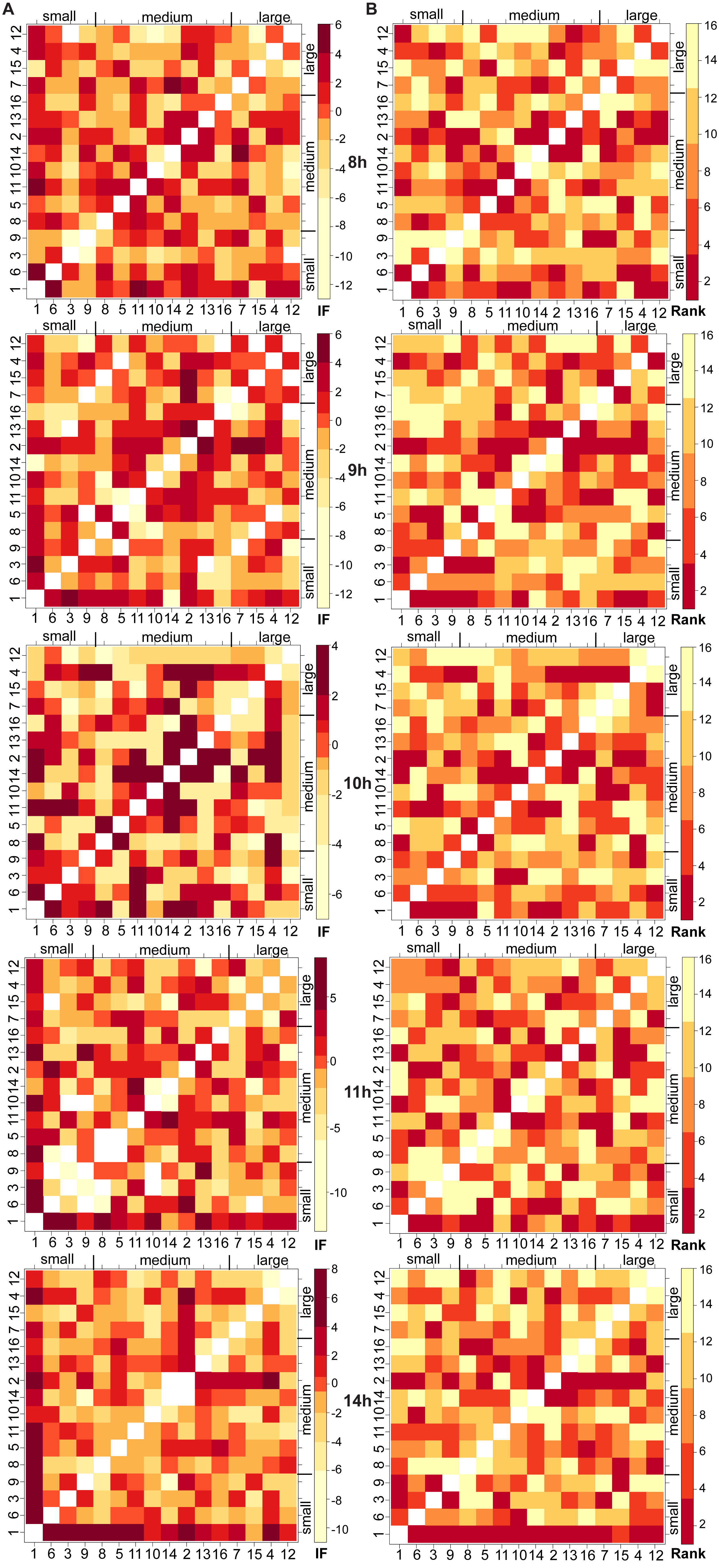

Supplement: S12 Fig — (A) Heatmap of normalized interaction values between non-homologous centromeres at each time point (8h, 9h, 10h, 11h and 14h). Centromeres are arranged from left to right and bottom to top according to their respective chromosome length, from shortest to longest. Darker shades of red indicate a higher level of interaction between non-homologous centromeres. Please note the log2 scale on the color key for interaction frequencies. (B) Heatmaps of ranked interaction frequencies between non-homologous centromeres at each time point (8h, 9h, 10h, 11h and 14h). Centromeres are arranged from left to right and bottom to top according to their respective chromosome length, from shortest to longest. For each centromere, darker shades of red indicate a rank closer to 1 for that interaction (strongest). (TIF) [file pgen.1006347.s012.tif]

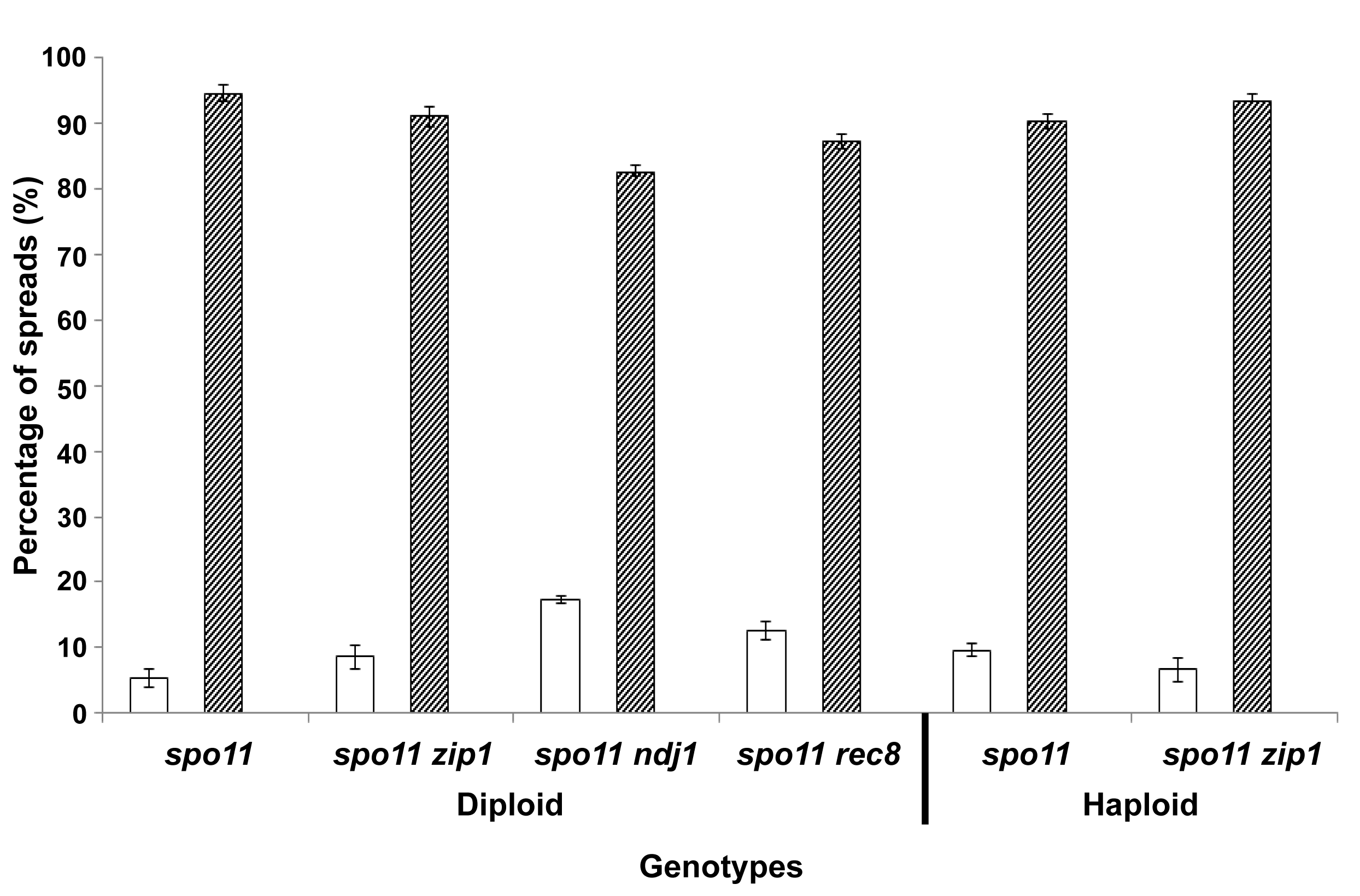

Supplement: S13 Fig — An aliquot of the cultures used for 3C2D-qPCR was taken to determine the centromere organization (Ctf19) by chromosome spreading. Spreads were classified as either separated/coupled centromeres (lined bars), or clustered centromeres/other status (plain bars), similarly to previous reports [17, 44]. About 50 individual spreads were assessed per independent replicate. The percentages of spreads in the two categories are given on the y-axis (mean +/- standard deviation), for multiple haploid and diploid yeast strains of various genotypes. (TIF) [file pgen.1006347.s013.tif]

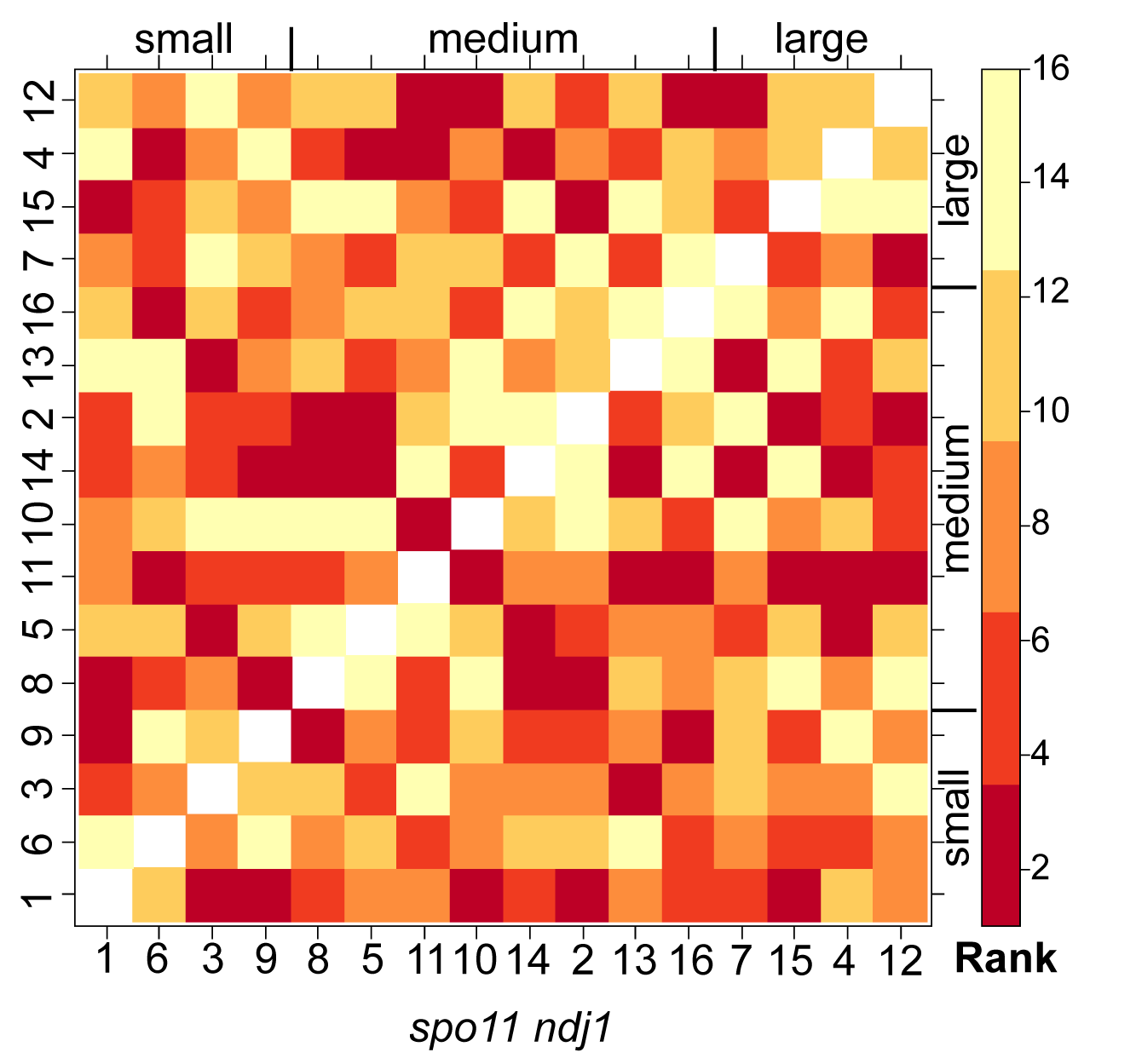

Supplement: S14 Fig — Centromeres are arranged from left to right and bottom to top according to their respective chromosome length, from shortest to longest. For each centromere, darker shades of red indicate a rank closer to 1 for that interaction (strongest). (TIF) [file pgen.1006347.s014.tif]

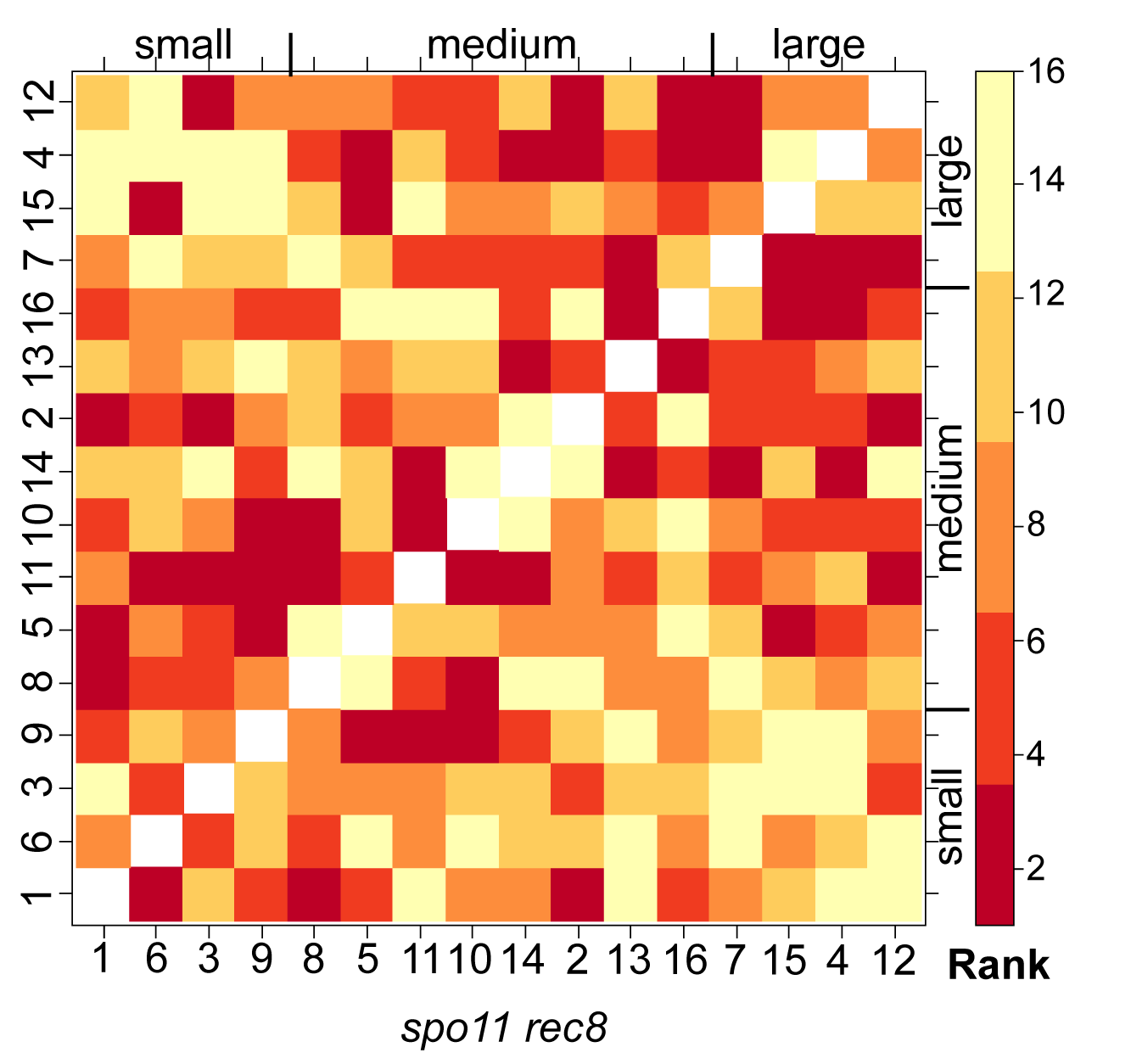

Supplement: S15 Fig — Centromeres are arranged from left to right and bottom to top according to their respective chromosome length, from shortest to longest. For each centromere, darker shades of red indicate a rank closer to 1 for that interaction (strongest). (TIF) [file pgen.1006347.s015.tif]
