# Supplementary material for: Multiple Pairwise Analysis of Non-homologous Centromere Coupling Reveals Preferential Chromosome Size-Dependent Interactions and a Role for Bouquet Formation in Establishing the Interaction Pattern
Source: PLoS Genet. 2016 Oct 21;12(10):e1006347. doi: 10.1371/journal.pgen.1006347 (PMC5074576; doi:10.1371/journal.pgen.1006347)
Supplement: S1 Table — (DOC) [file pgen.1006347.s016.doc]

**Table S1.** Yeast strains used in this study.

| **Strain** | **Genotype** | **Parent strain** | **Source** |
| --- | --- | --- | --- |
| BR1919-8B | Haploid MAT **α** leu2-3,112 his4-260,519 trp1-289 ura3-1 thr1-4 ade2-1 | - | Rockmill and Roeder, 1990 |
| PL140-1 | Diploid MAT **α**/MAT **a** leu2-3,112/ leu2-3,112 his4-260,519/ his4-260,519 trp1-289/ trp1-289 ura3-1/ ura3-1 thr1-4/ thr1-4 ade2-1/ade2-1 CTF19-13XMyc-KanMX4/ CTF19-13XMyc-KanMX4 | BR1919-8B | This study |
| TT714 | Same as PL140-1 but *spo11*::ADE2/*spo11*::ADE2 | BR1919-8B | Tsubouchi and Roeder, 2005 |
| TT708 | Same as TT714 but *zip1*::LEU2/*zip1*::LEU2 | BR1919-8B | Tsubouchi and Roeder, 2005 |
| PL141-1 | Same as TT714 but *ndj1*::HphMX4/*ndj1*::HphMX4 | BR1919-8B | This study |
| PL142-1 | Same as TT714 but *rec8*::HphMX4/*rec8*::NatMX4 | BR1919-8B | This study |
| TT892 | Same as BR1919-8B but thr1:MAT **a** THR1 and CTF19-13XMyc-KanMX4 | BR1919-8B | Tsubouchi and Roeder, 2005 |
| PL125-1 | Same as TT892 but *spo11*::ADE2 | BR1919-8B | This study |
| TT893 | Same as PL125-1 but *zip1*::LEU2 | BR1919-8B | Tsubouchi and Roeder, 2005 |
| PL136-2 | Same as PL125-1 but *ndj1*::HphMX4 | BR1919-8B | This study |
| PL137-2 | Same as PL125-1 but *rec8*::HphMX4 | BR1919-8B | This study |
| BR5873-18D | Same as PL125-1 but ura3:LacI-GFP-URA3, CEN3:LacO-LEU2, CEN5:TetO-URA3 and leu2:TetR-mCherry-HphMX4 | BR1919-8B | This study |
| BR5882-2D | Same as PL125-1 but ura3:LacI-GFP-URA3, CEN3:LacO-LEU2, CEN1:TetO-URA3 and leu2:TetR-mCherry-HphMX4 | BR1919-8B | This study |
