# Supplementary material for: Multiple Pairwise Analysis of Non-homologous Centromere Coupling Reveals Preferential Chromosome Size-Dependent Interactions and a Role for Bouquet Formation in Establishing the Interaction Pattern
Source: PLoS Genet. 2016 Oct 21;12(10):e1006347. doi: 10.1371/journal.pgen.1006347 (PMC5074576; doi:10.1371/journal.pgen.1006347)
Supplement: S2 Table — (DOC) [file pgen.1006347.s017.doc]

**Table S2. Primer sequences used for 3C-qPCR.**

| **Primer name** | **Sequence** |
| --- | --- |
| Chr8Base0kb | GTCATAGTACCTTTACCAAGATGCAATA |
| Chr8Short10kb | TGTCTGAAAACCTTTGTCAGTAAAATC |
| Chr8Long80kb | CACTCTTATTGAAGAAGGGGTAGACTC |
| MfeCEN1Lb | caagatcctagaagtgtgactggacgta |
| EcoCEN1R | CTAAATACAAACCTGCAAGGTCCTCATC |
| MfeCEN2L | CTATTGGCACCGACTCTGCTAGTATTTC |
| EcoCEN2Rd | ttagtcattcttcctatgcactagaccac |
| MfeCEN3L | CGGAGTATGGACATTATAAACGTGTGAA |
| MfeCEN3Rb | gtaggaaggtgatgatatgctccgtaaa |
| MfeCEN4L | TTTATCCTCAAAATGAATAGCTCCCCTA |
| MfeCEN4R | TCCGGTTTTATCGTCACAGTTTTACAGT |
| EcoCEN5L | GCATGTAGGAGAGATGTATTTTTGCTTG |
| MfeCEN5R | TGCTCACTACTGACTCACTGATCCTTTT |
| MfeCEN6L | TGTCAAACCTACGGTTCTTCAATACAGA |
| MfeCEN6R | CACAAAGAAGTAAACGATATGCTGGAGA |
| EcoCEN7Lc | aattacagtgttttcgtccgcacctt |
| EcoCEN7R | CGTCGGGGTAATGCATGTAAATAAATAG |
| EcoCEN8L | TTGTGGATGTGTAATTGTTGGAGTTCTT |
| MfeCEN8R | TTGGACCATCAATCAAAACCTATAGTAA |
| MfeCEN9L | AGAAGCGATAGTTTTTAAGCGGTCTTCT |
| MfeCEN9R | ATAGTTTACCCACAAGTTCATCCAGCTC |
| MfeCEN10L | TCAAAGAGAGTAACGATGAAACCTACGA |
| MfeCEN10R | TCGTTAATTTATCTGTCACACGGTTAGC |
| MfeCEN11L | ATTCATCCTTTTTCTTGTCATCATCGTT |
| EcoCEN11Rb | aagtcatcgtcaggattaccgtgaaa |
| MfeCEN12L | AAAGGAAAGGAAGTAGATGATGGCAAAT |
| EcoCEN12R | AGGTCAAAAGCCAACTCAGGTACAAATA |
| MfeCEN13Lb | tcggcatcaattttatttcccagtataa |
| MfeCEN13Rb | atcaatggactggtaatttcgtattcgt |
| MfeCEN14Lb | taaatctactcctcaccatcgtcttcatc |
| EcoCEN14R | CGAAGCCTCAGTTAGACCATAGTATTGC |
| MfeCEN15L | TAAACTCAAAGTCAAGAGGTTCCGATCT |
| MfeCEN15R | AGTTCTCAAGCTCATCTCGAAGTGTTTT |
| EcoCEN16Lb | acgagttgaaggatggaaagtacaatga |
| MfeCEN16R | CAGAATCAATGTTATGGCTGTTGATGAC |
| 3CinternalF | tggacgcacttcttgcatac |
| 3CinternalR | gacagctattttgcggcttc |
