# Supplementary material for: Multiple Pairwise Analysis of Non-homologous Centromere Coupling Reveals Preferential Chromosome Size-Dependent Interactions and a Role for Bouquet Formation in Establishing the Interaction Pattern
Source: PLoS Genet. 2016 Oct 21;12(10):e1006347. doi: 10.1371/journal.pgen.1006347 (PMC5074576; doi:10.1371/journal.pgen.1006347)
Supplement: S3 Table — (DOC) [file pgen.1006347.s018.doc]

**Table S3. Taqman probes used for 3C-qPCR.**

| **Probe name** | **Sequence** |
| --- | --- |
| Probe1CEN1L | cacgacgtgtcccttataaatcagttgtga |
| Probe2CEN1R | tgcgttctcgctcttataagcattctcagt |
| Probe3CEN2L | aaagtgctctttaatgggctttctctgatttt |
| Probe4CEN2R | tcgtaccaagccggttcacctttg |
| Probe5CEN3L | acagcttcgaacctttctgcataattacta |
| Probe6CEN3R | cggagctcacctattacttttgttagtattgtca |
| Probe7CEN4L | ccacaagaaattgcatacagcggtaaa |
| Probe8CEN4R | tgatacttgcttatctcatagttaactggcataaat |
| Probe9CEN5L | catcaagcccattcaatgcagatgt |
| Probe10CEN5R | cttaataggtgttgcccaactattcgt |
| Probe11CEN6L | atcgtgcatgaatacataccgcata |
| Probe12CEN6R2 | TCGGACGATTCCAAGAGTTCATTCAA |
| Probe13CEN7L | aggaacaacaacatcaagaagcctttca |
| Probe14CEN7R | aaaattgaatccttagcaggcatacat |
| Probe15CEN8L | acgcacgagcgaattaacattccta |
| Probe16CEN8R | tttcctgttttcaaagtatttcaatttactaacaac |
| Probe17CEN9L | ccgaaattattctccgctacttatatgc |
| Probe18CEN9R | caaccttaaagactgaaatttcgaacaataaaa |
| Probe19CEN10L | tggacccataatcatcatcgtgaa |
| Probe20CEN10R | aatcacctaataaaatagtatggccgtgaca |
| Probe21CEN11L | tcagaacatgctaaagtaaaccaaactgcta |
| Probe22CEN11R | agttgcaaagcacgcctcaaaatct |
| Probe23CEN12L | aacaagaaagtgaccttatttgccatca |
| Probe24CEN12R | agaaattctactggtccccgtcctaca |
| Probe25CEN13L | tttccaatcactgggtgttgctttc |
| Probe26CEN13R3 | TTGTTACCGTAGATTAAACCAATATTAAAGTAGAAA |
| Probe27CEN14L | tctaaaatacccgctccccgatctt |
| Probe28CEN14R | acgagttatttaagttggtctccaccattt |
| Probe29CEN15L | aattctttcctgaaaattcgcctaaatactga |
| Probe30CEN15R | caggtagtatataagagaataacttccctcttttgc |
| Probe31CEN16L | caaattctaccagtcgttcttggaataccatt |
| Probe32CEN16R | aatgtggcaatttccttccacgaat |
